# Cyclic Metallophosphines via an Unexpected Series of Metal-Mediated P–C and C–C Bond Rearrangements

**DOI:** 10.1021/acs.inorgchem.5c03677

**Published:** 2025-09-22

**Authors:** Mihir L. Bhowmik, Md. Abdullah Al Mamun, Vladimir N. Nesterov, Jagodish C. Sarker, Shariff E. Kabir, Graeme Hogarth

**Affiliations:** † Department of Chemistry, Jahangirnagar University, Savar, Dhaka 1342, Bangladesh; ‡ Department of Chemistry, Comilla University, Cumilla 3506, Bangladesh; § Department of Chemistry, 3404 University of North Texas, 1155 Union Circle, Box 305070, Denton, Texas 76203, United States; ∥ Department of Chemistry, Jagannath University, Dhaka 1100, Bangladesh; ⊥ Department of Chemistry, 4616 King’s College London, Britannia House, 7 Trinity Street, London SE1 IDB, U.K.

## Abstract

Thermal reactions of [Ru_3_(CO)_10_(μ-dppe)] (dppe = 1,2-bis­(diphenyl­phosphino)­ethane) and internal alkynes (RC≡CR, R = Ph, Et) afford 52-electron trinuclear clusters which are best considered as comprising an 18-electron cyclic metallo­phosphine ligand coordinated to a diruthenium center. They result from an unexpected and unprecedented series of metal-mediated ligand transformations, including the double P–Ph cleavage of one end of the diphosphine and capture of each of the resulting phosphinidine and benzyne ligands by the alkyne.

## Graphical abstract

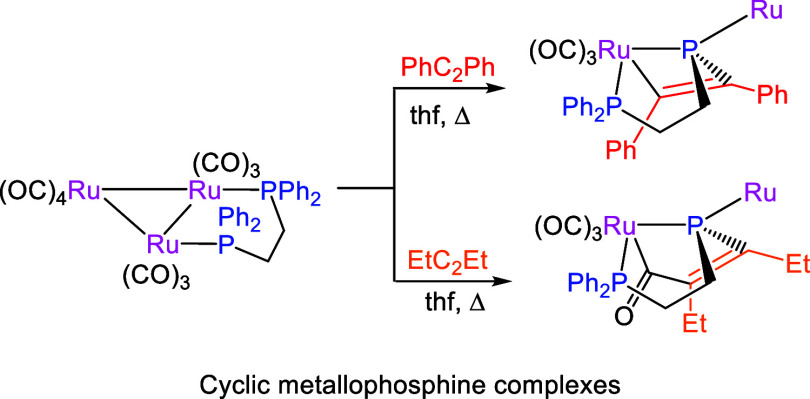

Phosphines are perhaps the most widely studied ligands in transition metal chemistry, being able to bind to metals in a wide range of oxidation states, and the ability to fine-tune both their electronic and steric properties means that they have been widely developed in homogeneous catalysis, being essential to many industrially important processes such as hydroformylation, cross-coupling, and hydrogenation. An interesting subset of phosphines comprises the (so-called) metallo­phosphines, whereby one of the substituents is a transition metal.^1–5^ Such a situation arises with a pyramidally bound metal–phosphido (PR_2_^–^) complex (Chart 1), the localized lone pair being available for coordination to a second metal center. Pyramidally bound metal–phosphido species contain an sp^3^-hybridized phosphorus,^6–10^ in contrast with planar phosphido ligands in which phosphorus is sp^2^-hydridized.^11^ Pyramidal phosphido ligands are often found at high-valent metal centers, where the lone pair and metal-based LUMOs are energetically disparate, and at low-valent metal centers, where the metal center is already coordinatively and electronically saturated and thus there is no low-lying LUMO for donation of the lone pair.

**Chart 1:**
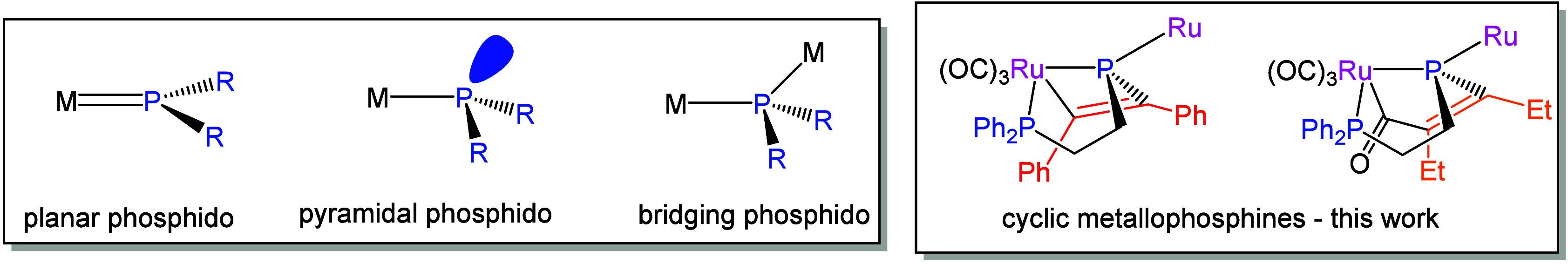
(a) Planar, Pyramidal, and Bridging Phosphido Ligands and (b) Cyclic Metallophosphines (Bridging Phosphido) Ligands Prepared in This Work

There are previous reports of pyramidal phosphido ligands bound to low-valent group 8 metal centers,^12^ some examples of which are given in Chart 2.^13–16^ Pyramidal phosphido ligands generally have two unconnected substituents, and these can be the same or different, the latter case conferring chirality of the phosphorus center. It is possible to link the two substituents and make cyclic phosphido ligands, but as far as we are aware, there are no previous examples of the metal center also being connected to form cyclic metallo­phosphines. In this Communication, we describe the serendipitous formation of two such cyclic metallo­phosphines (Chart 1) in which a low-valent, formally Ru­(II) center is incorporated into the cyclic structure. As prepared, the metallo­phosphines are coordinated to a diruthenium center that is bridged by a previously unknown organyl ligand formed via the C–C coupling of benzyne and a second (different) internal alkyne.

**Chart 2:**
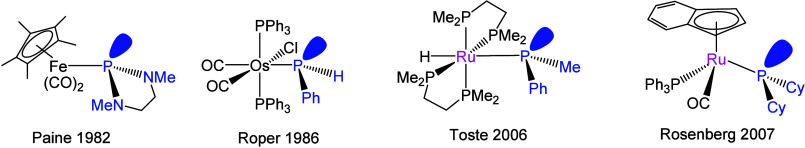
Examples of Previously Reported Low-Valent Group 8 Complexes Containing a Pyramidal Phosphido Ligand

Over many years, the reactivity of low-valent clusters toward alkynes has been extensively explored, reaction outcomes varying depending upon the nature of the alkyne.^17,18^ With internal alkynes (RC≡CR), typical products from reactions with [M_3_(CO)_12_] (M = Ru, Os) and their MeCN-substituted derivatives are alkyne-capped clusters [M_3_(CO)_10_(μ_3_-alkyne)] ^19–22^ along with complexes containing two or more alkyne ligands resulting from metal-mediated C–C coupling reactions.^17,18,23,24^ Fragmentation of the trinuclear framework of these clusters is often a competing process, and to stabilize the trinuclear center, diphosphines can be incorporated, with bis­(diphenyl­phosphino)­methane (dppm)^25^ and 1,2-bis­(diphenyl­phosphino)­ethane (dppe)^26^ being widely utilized in this regard. Their incorporation can, however, lead to further reaction pathways becoming accessible, primarily a result of C–H (ortho­metalation) and/or P–Ph bond scission, which can result in the formation of unusual metal-bound ligands that are otherwise often inaccessible by standard synthetic strategies.^27^ While the reactivity of [Ru_3_(CO)_10_(μ-dppm)] toward alkynes has been extensively studied,^27^ as far as we are aware, the related chemistry of [Ru_3_(CO)_10_(μ-dppe)] has been neglected. Herein we give preliminary details of our work in this area, focusing on the reactivity of [Ru_3_(CO)_10_(μ-dppe)] toward internal alkynes, diphenyl­acetylene and hex-3-yne, which reveals some quite unexpected and complex metal-mediated transformations that afford previously unknown cyclic metallo­phosphine complexes (Scheme 1).

**Scheme 1:**
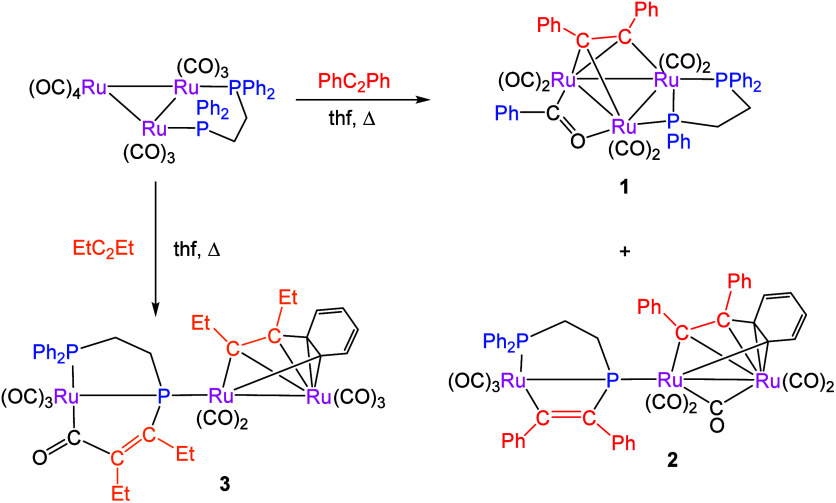
Isolated Products of Reactions of RC≡CR (R = Ph, Et) with [Ru_3_(CO)_10_(μ-dppe)]

Heating [Ru_3_(CO)_10_(μ-dppe)] and a slight excess of diphenyl­acetylene at 66 °C led to the formation of a mixture of two complexes, namely **1** (30%) and **2** (25%). The formation of the 48-electron cluster **1** was not unexpected,^27^ resulting from alkyne addition and oxidative addition of a single P–Ph bond, the resulting phenyl group being trapped at the cluster center as an acyl group following the migratory carbonyl insertion. In contrast, the formation of the open 52-electron cluster **2** was quite unexpected, and further, a similar reaction between [Ru_3_(CO)_10_(μ-dppe)] and a slight excess of hex-3-yne at 66 °C afforded the closely related 52-electron cluster **3** (15%) as the sole isolated reaction product. All new complexes have been fully characterized, including by single-crystal X-ray diffraction, the results of which are shown for **2**·CH_2_Cl_2_ and **3**·C_6_H_14_ in Figure 1.

**Figure 1:**
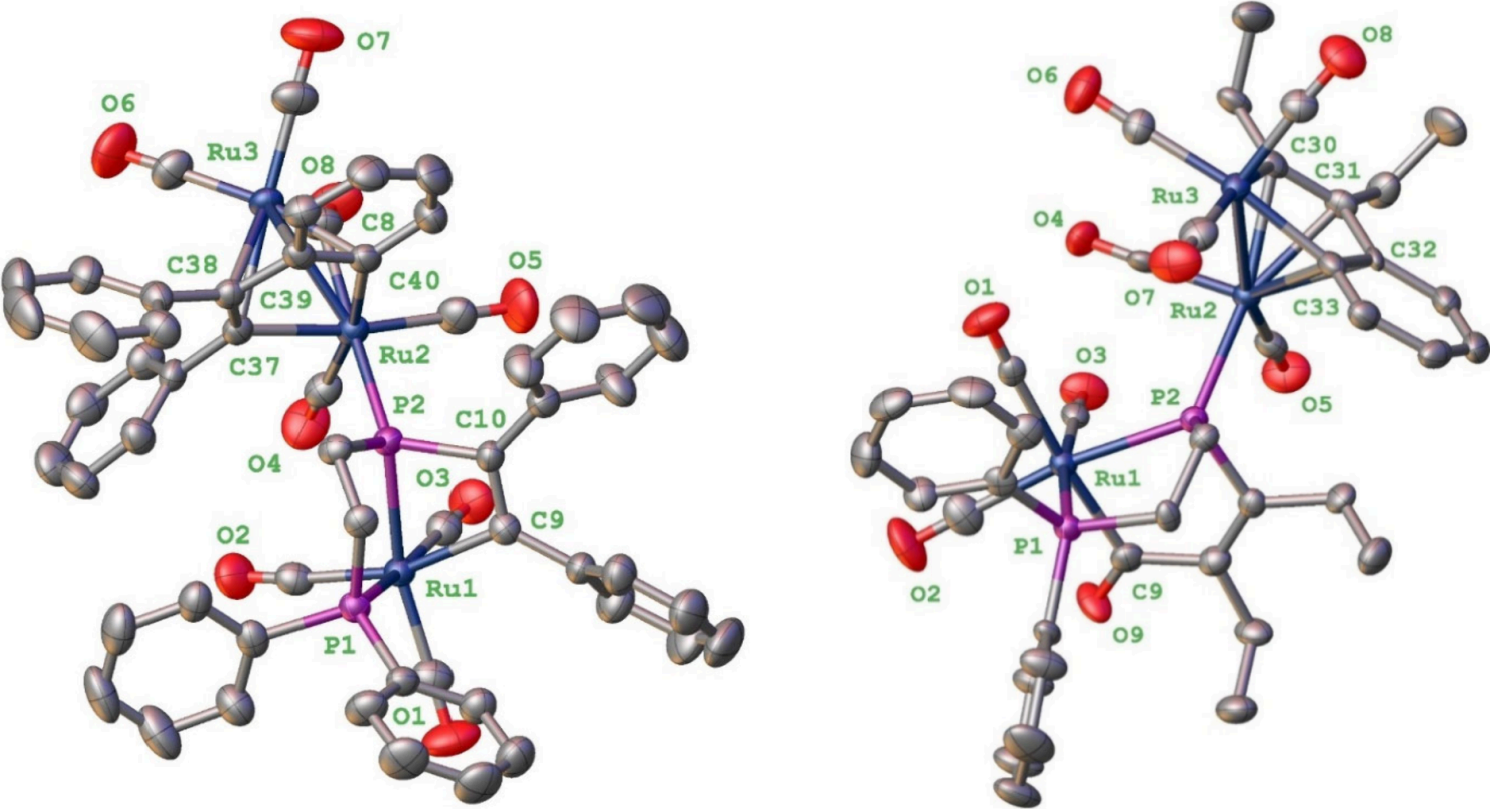
Molecular structures of **2** (left) and **3** (right) showing 50% probability of thermal ellipsoids, with hydrogen atoms and cocrystallized solvent omitted for clarity.

The molecular structures of compounds **2** and **3** are similar. Each contains a diruthenium center bridged by an organyl ligand which results from the fusion of an alkyne with benzyne, the latter resulting from a phenyl group being cleaved from the diphosphine and then orthometalated. The generation of benzyne is not unexpected; for example, heating [Ru_3_(CO)_11_(PPh_3_)] in toluene affords a mixture of [Ru_4_(CO)_11_­(μ_4_-PPh)­(μ_4_-η^4^-C_6_H_4_)] and [Ru_5_(CO)_13_­(μ_4_-PPh)­(μ_5_-η^6^-C_6_H_4_)],^28^ while heating [Ru_3_(CO)_10_­(μ-dppe)] at 120 °C for 16 h affords a mixture of [Ru_3_(CO)_9_­(μ-PhPCH_2_CH_2_PPh_2_)­(μ-Ph)] and the benzyne cluster [Ru_3_(CO)_9_­(μ-CO)­(μ-PCH_2_CH_2_PPh_2_)­(μ_4_-η^4^-C_6_H_4_)].^29,30^ Further, coupling of cluster-bound benzyne with other alkynes has been realized at a triosminum center.^31^ The organyl ligand in **2** and **3** lies perpendicular to the Ru–Ru vector and acts as a 6-electron donor. The Ru_2_(CO)_5_–(organyl) subunit is then characterized by 32 electrons, coordination of the metallo­phosphine affording the further 2 electrons to afford an electron-precise bimetallic containing a single Ru–Ru bond.

The metallophosphine coordinates to the diruthenium center, and the Ru(2)–P(2) bond lengths of 2.3585(11) and 2.3784(19) Å are within the expected range. The metallo­phosphine formally results from scission of two phenyl groups from one end of the diphosphine, an unexpected transformation but one previously noted.^29,30^ An equivalent of alkyne has added across the Ru–P bond in the putative {κ^2^-Ph_2_P­(CH_2_)_2_P}Ru moiety, and in **3**, migratory carbonyl insertion has also occurred into the generated Ru–C­(Et) bond, affording a less-ring-strained 5-membered metalla­cycle. We note that alkyne addition across terminal metal–phosphido ligands has been previously reported to afford 4-membered metallo­cycles M–PR_2_–C­(R)=C­(R),^10,32^ akin to that in **2**. Most closely related to the formation of **2** and **3** is a similar alkyne addition across a bridging phosphinidine ligand at a dimolybdenum center.^33^ This yields a Mo–PR–C­(R)=C­(R) ring that undergoes (reversible) migratory carbonyl insertion to afford a Mo–PR–C­(R)=C­(R)–C­(O) ring akin to that in **3**. The Ru(1)–P bond lengths are all similar, being close to those between Ru(2) and P(2), and the P(1)–Ru(1)–P(2) bond angles are 83.38(4) and 84.77(6)° in M–PR_2_–C­(R)=C­(R) and M–PR–C­(R)=C­(R)–C­(O), respectively. Thus, the overall geometry at Ru(1) in both is best described as a *fac* octahedral. The most significant deviation from octahedral geometry around Ru(1) in **2** is the C(1)–Ru(1)–C(9) angle of 99.41°, and this relaxes in **3** upon insertion of CO—probably why migratory insertion is favored here. The pyramidal nature of Ru(2) is shown by the Ru(2)–P(2)–X bond angles ranging from 113.14 to 125.89° in **2** and from 111.45 to 123.08° in **3**, the largest in each case being the Ru(2)–P(2)–Ru(1) angle. As the new tripodal ligands are 4-electron donors, then Ru(1) is an 18-electron center. Spectroscopic data support the solid-state structures—most notably, the ^31^P­{^1^H} NMR spectra show a pair of doublets at 53.7 and −38.5 (*J* = 58.8 Hz) in **2** and at 60.1 and −35.2 (*J* = 58.8 Hz) in **3**, the low-field resonance in each case being assigned to the phosphido phosphorus. The four distinct atoms bound to P(2) create a chiral center, but the molecule is isolated as a racemic mixture.

The overall stoichiometries relating to formation of **2** and **3** are quite straightforward, [Ru_3_(CO)_10_(μ-dppe)] reacting with 2 equiv of alkyne and resulting in a loss of benzene and either 4 (**2**) or 3 (**3**) equiv of CO. Their precise mode of formation is unclear but requires the following events: (i) a double P–Ph bond cleavage from one end of the diphosphine, likely to occur in a stepwise manner; (ii) orthometalation of one metal-bound phenyl group, which would then generate benzyne and a hydride, the latter eliminating benzene upon reaction with the second metal-bound phenyl; and (iii) reaction of both the generated phosphinidine and benzyne groups with 1 equiv of alkyne.

We have not attempted to remove the metallo­phosphines from the diruthenium center, and we suspect that quite forcing and/or oxidizing conditions would be required to do so, most likely resulting in elimination of the phosphine oxide. The isolation of **2** and **3** and their thermal and oxidative stability do, however, suggest that these and related cyclic metallo­phosphines would be stable and display a rich coordination chemistry, should suitable preparative routes be found. It is not easy to see how the metallo­phosphines in **2** and **3** could be prepared off metal, and indeed, building the M–C bond is always going to be a challenge. Related cyclic metallo­phosphines may be most easily achieved upon deprotonation of (R_2_PCH_2_CH_2_)_2_PH following coordination of all three phosphorus centers, akin to the known coordination behavior of bis­(2-diphenyl­phosphino­ethyl)­amine, (Ph_2_PCH_2_CH_2_)­NH,^34^ upon deprotonation.^35–37^ In this regard, we note the synthesis of trisphosphines, (R_2_PCH_2_CH_2_)_2_PH (R = Me, Et), and their subsequent deprotonation and coordination to ZrCl_4_,^38^ although no monomeric metallo­phosphine complexes were isolated. Our current efforts center on screening reactions of [Ru_3_(CO)_10_(μ-dppe)] with other alkynes and trying to isolate intermediates en route to **2** and **3**. These studies, along with a comparative study of the reactivity of [Ru_3_(CO)_10_(μ-dppm)] with internal alkynes,^27^ will be reported in due course.

## Supplementary Material



## Data Availability

Atomic coordinates for all optimized structures are available from VNN on request.

## Notes

Experimental details and characterizing data, ^1^H and ^31^P­{^1^H} NMR data, IR data, crystal data and structure refinement details, and selected bond lengths and angles (PDF)
