# Correction to “Cyclic Metallophosphines via an Unexpected Series of Metal-Mediated P–C and C–C Bond Rearrangements”

**DOI:** 10.1021/acs.inorgchem.6c04414

**Published:** 2026-10-08

**Authors:** Mihir L Bhowmik, Md. Abdullah Al Mamun, Vladimir N. Nesterov, Jagodish C. Sarker, Shariff E. Kabir, Graeme Hogarth

We erroneously reported that complexes **2** and **3** were prepared in refluxing THF, but the solvent used for the reaction was actually toluene. This does not affect any conclusions made in the paper. Further, in the Supporting Information, the ^1^H NMR spectrum of **3** (Figure S6) was incorrect. We now give the correct spectra in Figures S6–S8 and also another version of the ^31^P­{^1^H} NMR spectrum (Figure S9), recorded with the same sample as the ^1^H NMR spectrum. The ^1^H NMR spectrum of **3** is very complicated as each methylene proton is inequivalent and this leads to 12 different methylene proton sites in the complex. In addition, the text of Section 1.3 of the Supporting Information has been corrected. The changes are reflected in the Supporting Information file with this Correction.

## Supplementary Material

