# Supplementary material for: Cyclic Metallophosphines via an Unexpected Series of Metal-Mediated P–C and C–C Bond Rearrangements
Source: Inorg Chem. 2025 Sep 22;64(39):19520–4. doi: 10.1021/acs.inorgchem.5c03677 (PMC12505257; doi:10.1021/acs.inorgchem.5c03677)
Supplement: Supplementary file 1 [file ic5c03677_si_001.pdf]

Cyclic metallophosphines via an unexpected series of metal-mediated P–C and C–C bond rearrangements

Mihir L. Bhowmik[a,b], Md. Abdullah Al Mamun[a], Vladimir N. Nesterov[c], Jagodish C. Sarker[d,*], Shariff E. Kabir[a, d,*] and Graeme Hogarth[e,*]

[a] *Department of Chemistry, Jahangirnagar University, Savar, Dhaka 1342, Bangladesh*
[b] *Department of Chemistry, Comilla University, Cumilla 3506, Bangladesh*
[c] *Department of Chemistry, University of North Texas, 1155 Union Circle, Box 305070, Denton,TX 76203, USA*
[d] *Department of Chemistry, Jagannath University, Dhaka 1100, Bangladesh*
[e] *Department of Chemistry, King's College London, Britannia House, 7 Trinity Street, London SE1 IDB, UK.*

*Corresponding authors, Email: graeme.hogarth@kcl.ac.uk (GH), jagodish@chem.jnu.ac.bd (JCS), skabir_ju@yahoo.com (SEK)

Supplementary Information

1.1. General Data

Unless otherwise stated, all reactions and manipulations were performed under a nitrogen atmosphere using standard Schlenk techniques. Reagent-grade solvents were dried by standard methods and were freshly distilled before use. $[Ru_3(CO)_{12}]$ was purchased from Strem Chemicals Inc. and diphenylacetylene, hex-3-yne and dppe from Sigma–Aldrich and used without further purification. $[Ru_3(CO)_{10}(\mu\text{-dppe})]$ was prepared according to the published procedure [1]. Infrared spectra of CH_2Cl_2 solutions were recorded on a Shimadzu FTIR Prestige 21 spectrophotometer over the range 2200–1600 cm^{-1}. Solution NMR spectra were recorded at room temperature on a Bruker Avance III HD (400 MHz) spectrometer. Chemical shifts (δ) were referenced internally to the residual solvent proton resonance (^1H) or external 85% H_3PO_4 (^{31}P). Elemental analyses were done by the Microanalytical Laboratories of the Wazed Miah Science Research Center at Jahangirnagar University. Preparative TLC separations were carried in the air on 20 x 20 cm^2 TLC plates coated with 0.25 mm of silica gel (HF254-type 60, E. Merck, Germany).

1.2. Reaction of $[Ru_3(CO)_{10}(\mu\text{-dppe})]$ with PhC≡CPh

A mixture of $[Ru_3(CO)_{10}(\mu\text{-dppe})]$ (50 mg, 0.051 mmol) and PhC≡CPh (36 mg, 0.202 mmol) in thf (15 mL) was heated to reflux at 66 °C for 9 h. The solvent was removed under reduced pressure and the residue chromatographed by TLC on silica gel. Elution with cyclohexane/CH_2Cl_2 (7:3, v/v) developed four bands. The first was unreacted starting material (trace), the second gave an

uncharacterized product as a green powder (trace), the third afforded **1** (16.4 mg, 30%) as red crystals, while the fourth yielded **2** (15.3 mg, 25%) as orange crystals after recrystallization from hexane/CH_2Cl_2 at –4 °C. Analytical and spectroscopic data for **2**: Anal. Calcd. for $C_{56}H_{38}O_8P_2Ru_3$: C, 55.86; H, 3.18. Found: C, 56.05; H, 3.35%. IR (ν(CO), CH_2Cl_2): 2086 vs, 2027 s, 2000 vs, 1967 m, 1942 w, 1842 br cm^{-1}. ^1H NMR ($CDCl_3$): δ 7.86 (m, 1H), 7.56 (m, 3H), 7.40 (m, 5H), 7.20 (m, 4H), 7.09 (m, 4H), 7.03 (m, 3H), 6.95 (m, 5H), 6.81 (m, 2H), 6.62 (m, 1H), 6.54 (d, J 7.6 Hz, 3H), 6.39 (m, 3H), 2.96 (m, 1H), 2.48 (m, 1H), 2.31 (m, 1H), 2.19 (m, 1H). $^{31}P\{^1H\}$ NMR ($CDCl_3$): δ 53.7 (d, J 58.8 Hz, 1P), –38.5 (d, J 58.8 Hz, 1P).

1.3. Reaction of [Ru_3(μ-dppe)$(CO)_{10}$] with EtC≡CEt

A thf solution (20mL) of [Ru_3(μ-dppe)$(CO)_{10}$] (40 mg, 0.04 mmol) and EtC≡CEt (13 mg, 0.158 mmol) was heated to reflux for 8 h. The solvent was removed under reduced pressure and the residue chromatographed by TLC on silica gel. Elution with cyclohexane/CH_2Cl_2 (7:3, v/v) developed three bands. The first gave a trace amount of an uncharacterized red powder, the second furnished **3** (6.4 mg, 15%) as orange crystals after recrystallization from hexane/CH_2Cl_2 at –4°C. Analytical and spectroscopic data for: Anal. Calcd. for $C_{41}H_{38}O_9P_2Ru_3$: C, 47.36; H, 3.68. Found: C, 47.88; H, 3.95%. IR (ν(CO), CH_2Cl_2): 2094 s, 2042 s, 2011m, 1980 vs, 1760 m cm^{-1}. ^1H NMR ($CDCl_3$): δ 7.56 (m, 2H), 7.40 (m, 2H), 7.22–6.92 (m, 5H), 6.82 (m, 2H), 6.55 (d, J 7.6 Hz, 3H), 2.92 (m, 2H), 2.34 (m, 2H), 2.60–2.74 (m, 8H), 1.05–1.19 (12H). $^{31}P\{^1H\}$ NMR ($CDCl_3$): δ 60.1 (d, J 58.8 Hz, 1P), –35.2 (d, J 58.8 Hz, 1P).

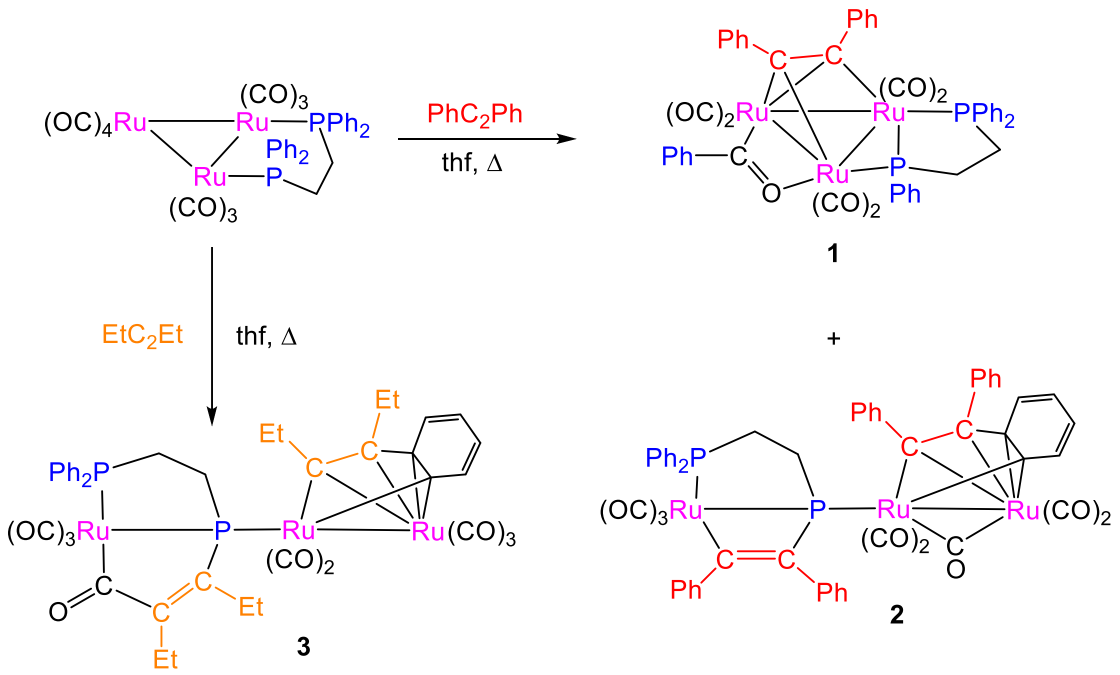

Scheme S1. Isolated products of reactions of RC≡CR (R = Ph, Et) with [$Ru_3(CO)_{10}$(μ-dppe)]

1.4. X-ray Crystallography

Single crystals of **2−3** suitable for X-ray diffraction analysis were grown by slow diffusion of hexane into a CH_2Cl_2 solution of the compounds at 4 °C. Each suitable single crystal was mounted on a Bruker APEX3 microsource diffractometer using a Nylon loop and Paratone oil using Mo-Kα radiation (λ = 0.71073). The diffraction data were collected at 253 K (for **2**) and 213 K (for **3**). Unit cell determination, data reduction, and absorption correction were done with SAINT V8.38A [2], and absorption corrections were applied using the program SADABS [3]. All the structures were solved by using the SHELXT [4] structure solution program and refined by full-matrix least-squares on F^2 using SHELXL [5] within the OLEX2 [6] graphical user interface. In all structures, non-hydrogen atoms were refined anisotropically, and the hydrogen atoms were included using a riding model. Relevant crystallographic parameters are given in Table 1.

References

[1] M.I. Bruce, T.W. Hambley, B.K. Nicholson, M.R. Snow, Cluster chemistry: X. Preparation of 1,2-bis(diphenylphosphino)ethane derivatives of $Ru_3(CO)_{12}$: crystal and molecular structures of $Ru_3(CO)_{10}(\mu\text{-}Ph_2PCH_2CH_2PPh_2)$, J. Organomet. Chem. 235 (1982) 83–91, https://doi.org/10.1016/S0022-328X(00)85723-X.

[2] Bruker, SAINT (V8.38A), Bruker AXS Inc., Madison, Wisconsin, USA, 2018.

[3] Bruker, SADABS-2016/2, Bruker AXS Inc., Madison, Wisconsin, USA, 2016..

[4] Sheldrick, G.M. (2015). Acta Cryst. A71, 3-8.

[5] G. M. Sheldrick, Crystal structure refinement with SHELXL, Acta Crystallogr. C71 (2015) 3–8, doi:10.1107/S2053229614024218.

[6] O.V. Dolomanov, L. Bourhis, R.J. Gildea, J.A.K Howard, H. Puschmann, OLEX2: a complete structure solution, refinement and analysis program, J. Appl. Crystallogr. 42 (2009) 339–341, https://doi.org/10.1107/S0021889808042726n.

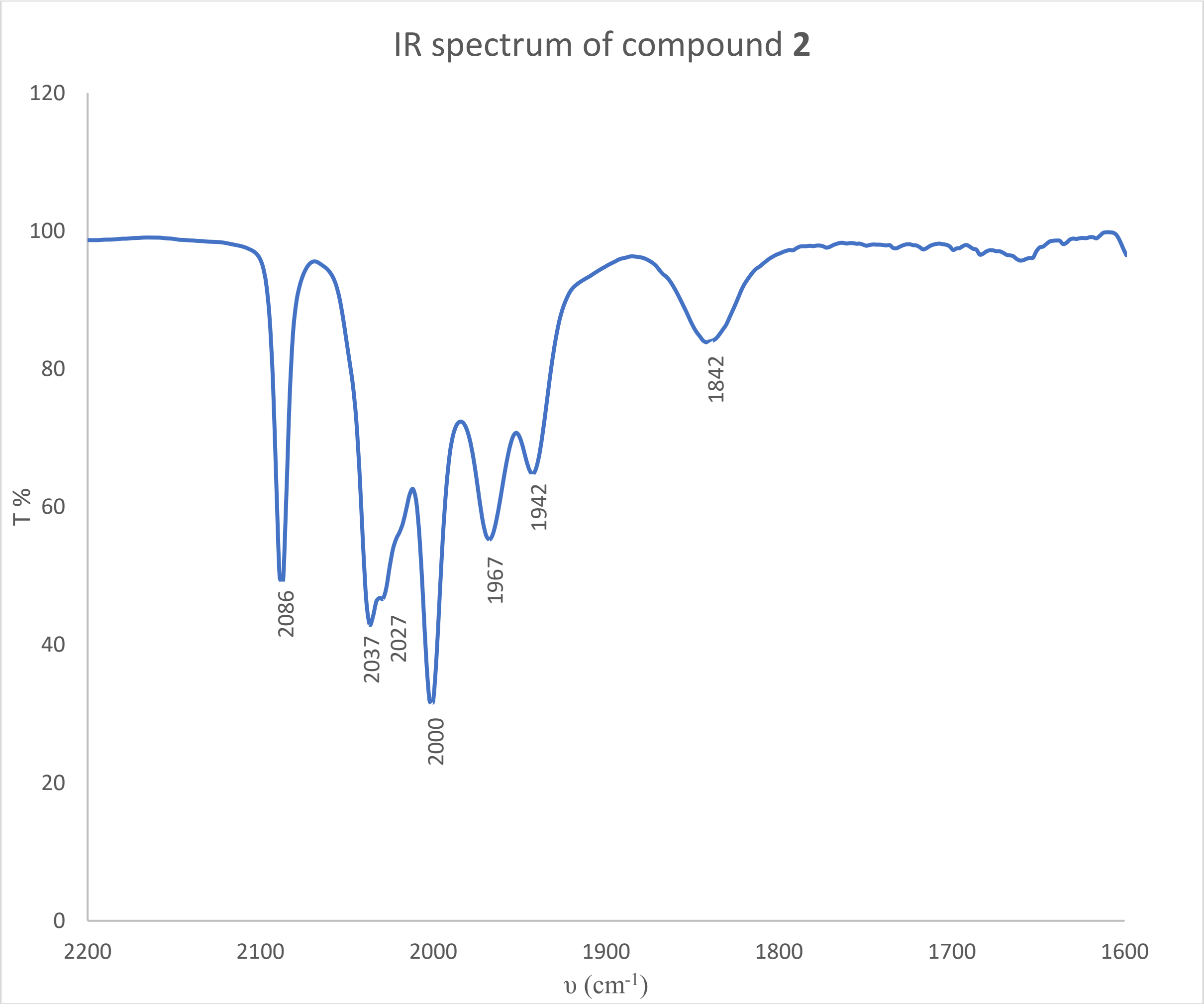

Fig. S1 IR spectrum of **2**

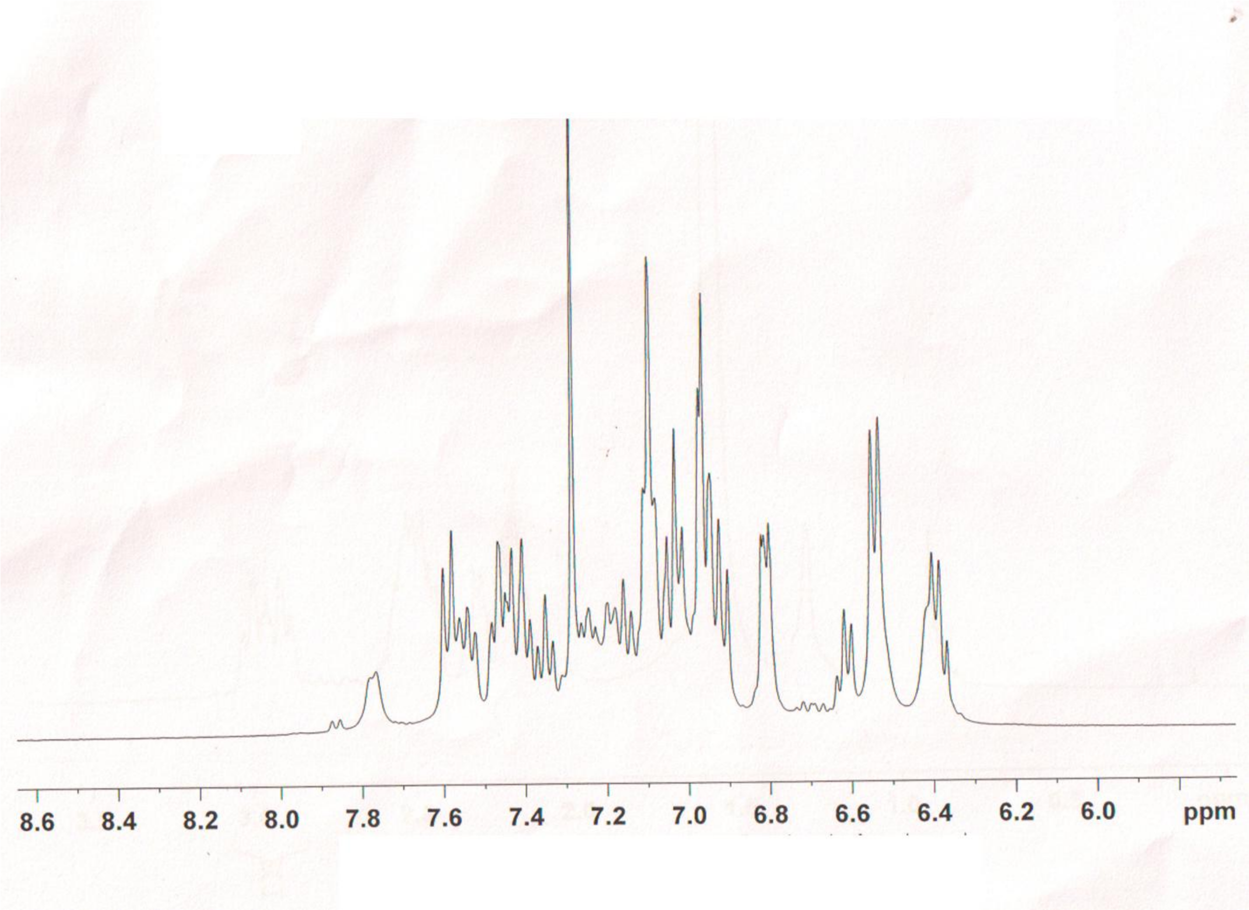

Fig. S2 ^{1}H NMR spectrum of **2 (**aromatic region).

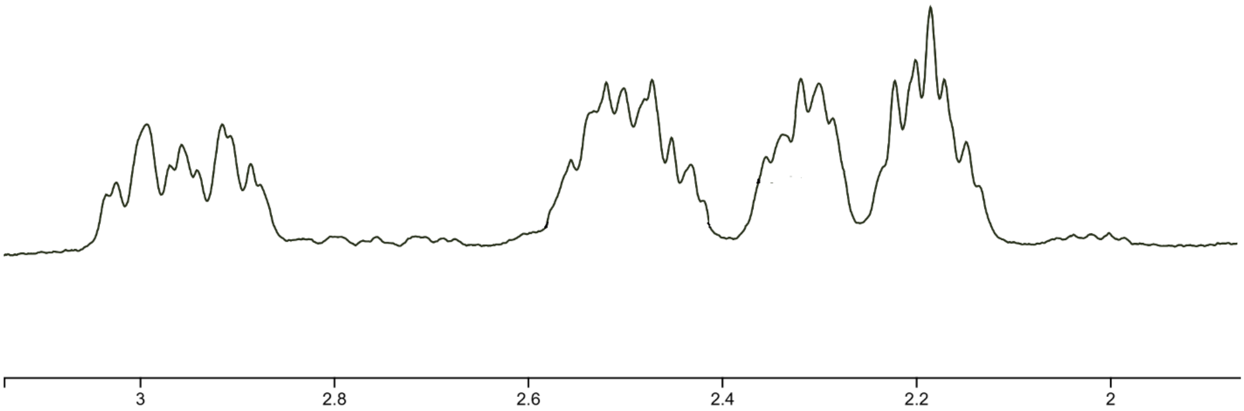

Fig. S3 ^{1}H NMR spectrum of **2 (**aliphatic region).

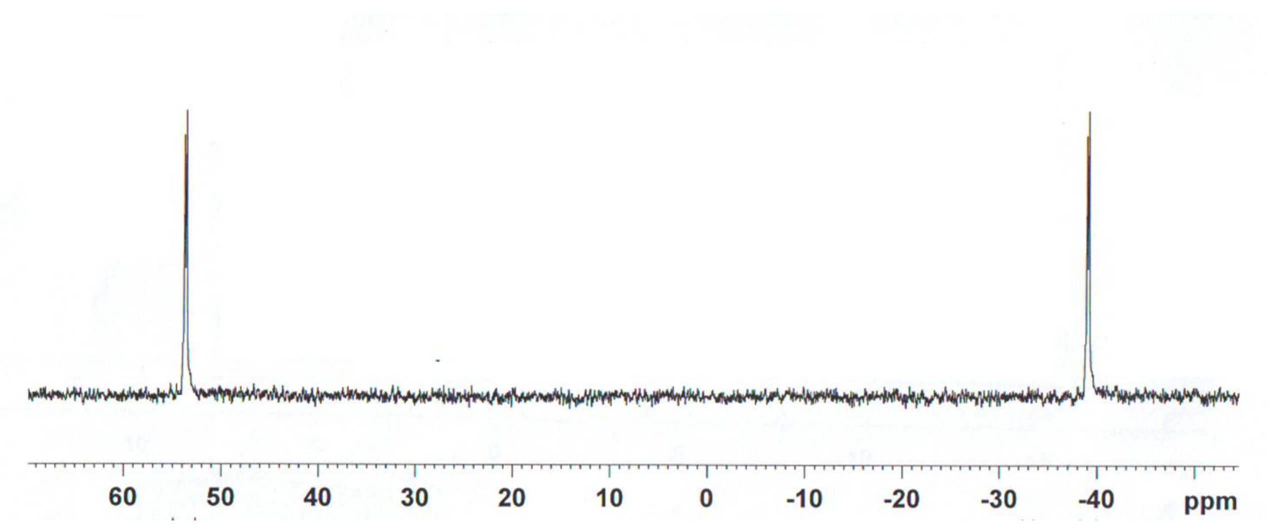

Fig. S4 $^{31}P\{^1H\}$ NMR spectrum of **2**.

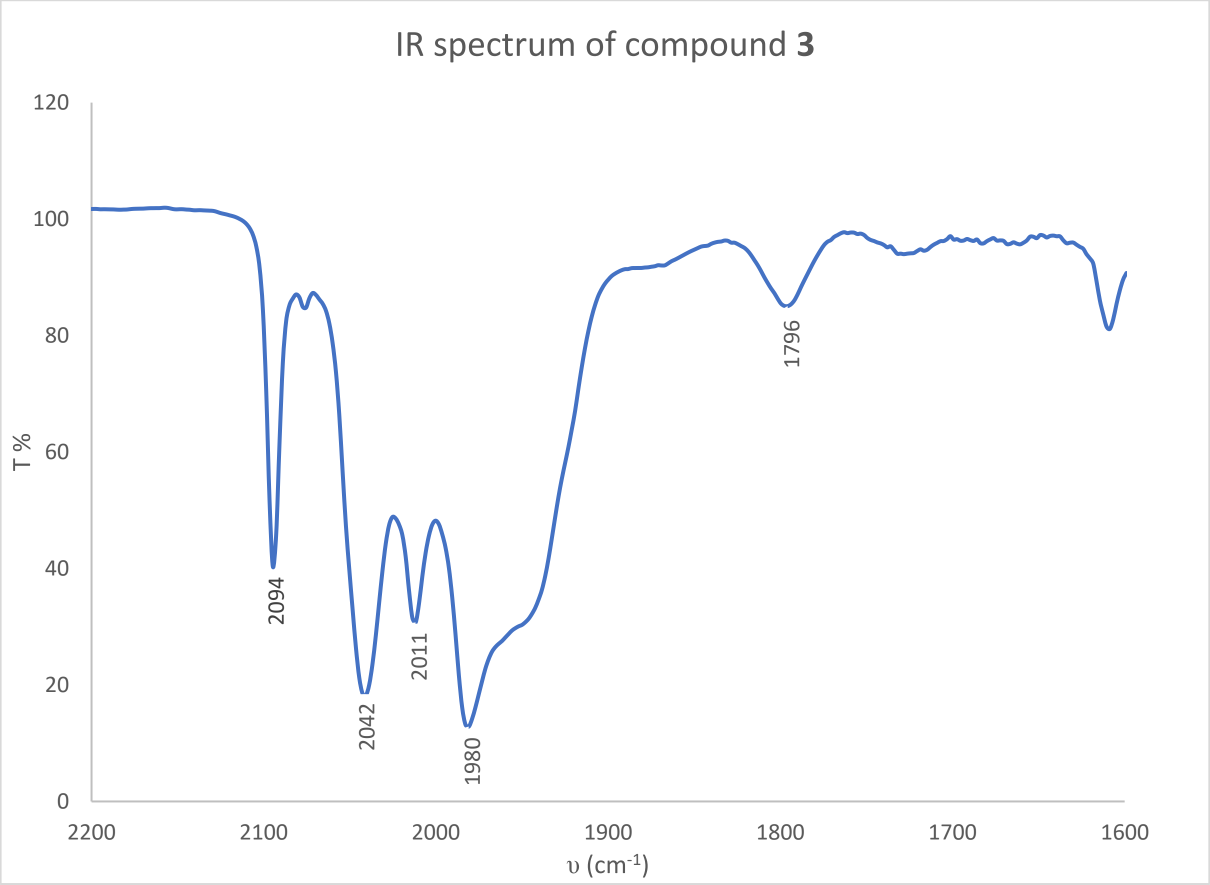

Fig. S5 IR spectrum of **3**

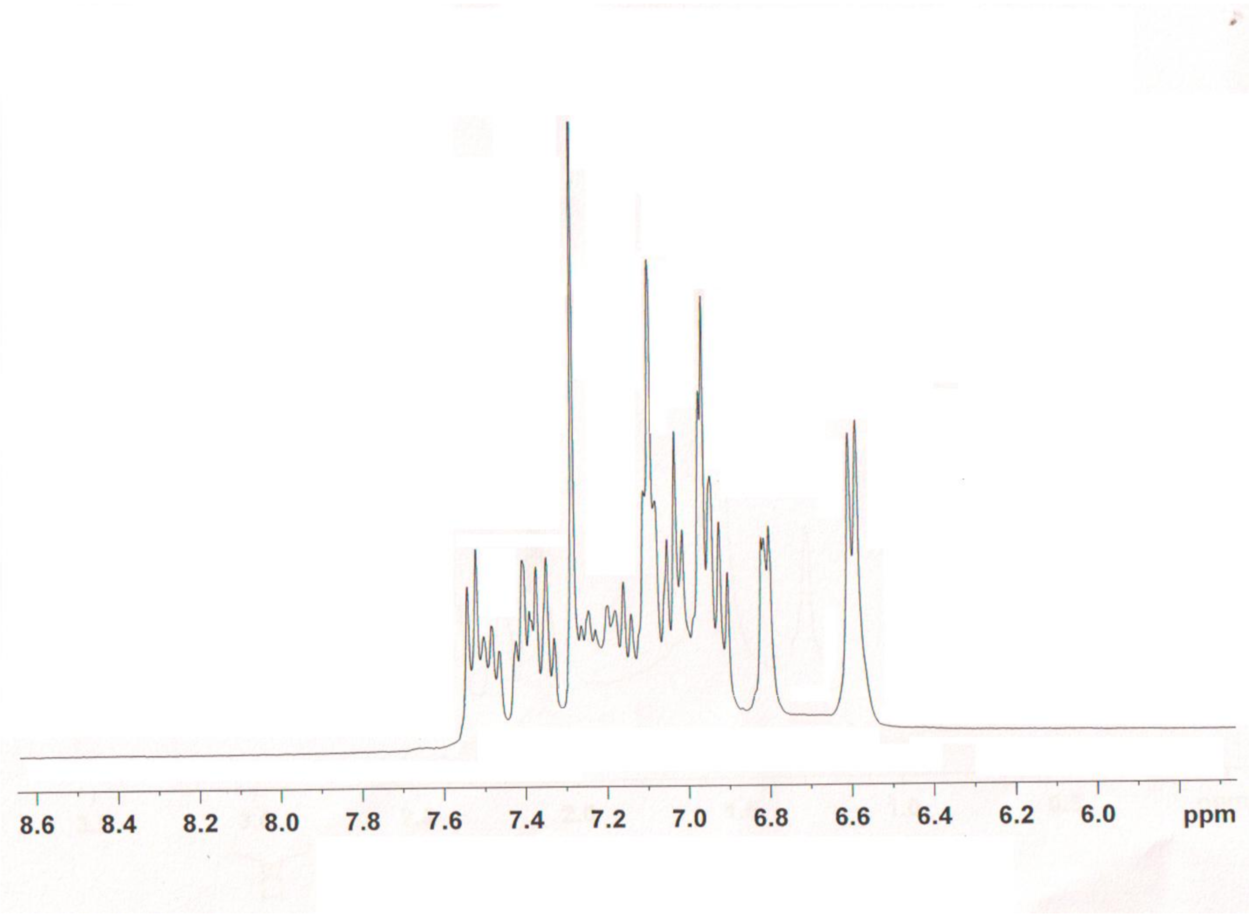

Fig. S6 ^{1}H NMR spectrum of **3** (aromatic region).

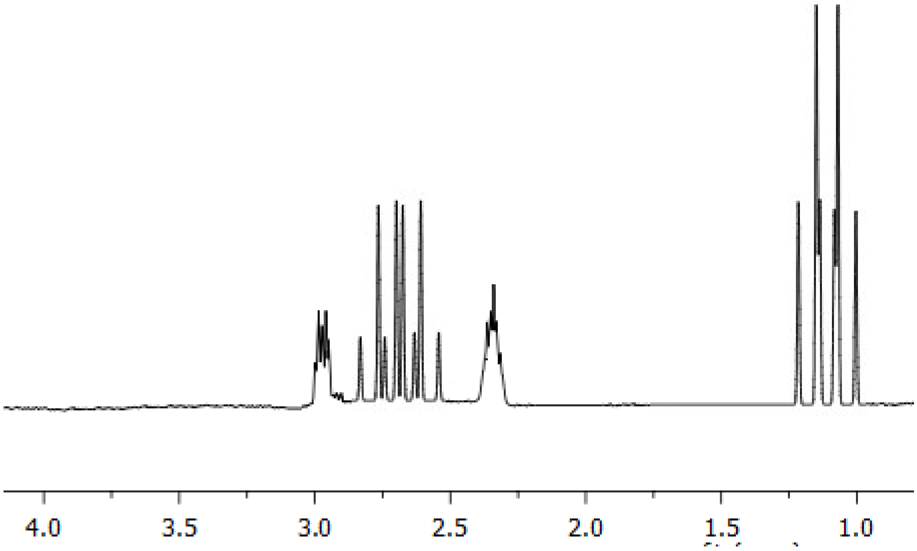

Fig. S7 ^{1}H NMR spectrum of **3** (aliphatic region).

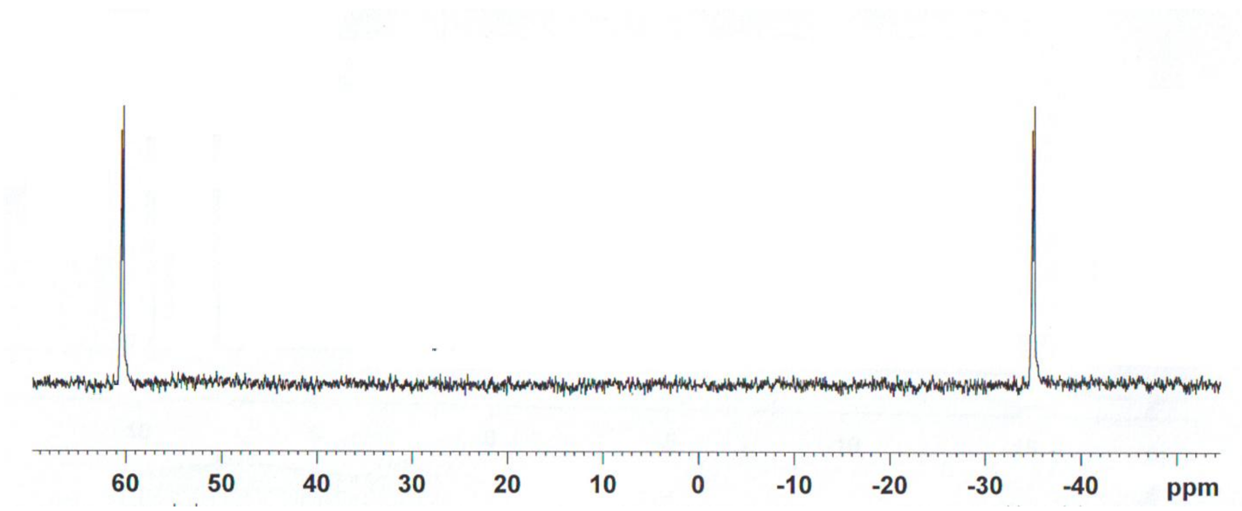

Fig. S8 $^{31}P\{^{1}H\}$ NMR spectrum of **3**.

Table S1. Crystal data and structure refinement details for **2–3**.

**Compound**	**2**	**3**
CCDC	2453401	2453416
Empirical formula	$C_{56}H_{38}O_8P_2Ru_3$,3CH2Cl2	$C_{41}H_{38}O_9P_2Ru_3$, 0.5C6H14
Formula weight	1458.79	1082.95
Temperature (K)	253.0	213.0
Wavelength (Å)	0.71073	0.71073
Crystal system	Monoclinic	Triclinic
Space group	P 2/c	P-1
Unit cell dimensions:		
a (Å)	21.4024(9)	10.0575(4)
b (Å)	14.6247(6)	13.4781(5)
c (Å)	19.8990(8)	17.3208(7)
α (°)	90	79.432(2)
β (°)	106.781(1)	76.337(2)
γ (°)	90	72.664(2)
Volume (Å^3)	5963.2(4)	2161.6(2)
Z	4	2
Density (calculated) (Mg/m^3)	1.625	1.664
Absorption coefficient (mm^{-1})	1.125	1.163
F(000)	2904.0	1086.0
Crystal size (mm^3)	0.401 × 0.255 × 0.031	0.088 × 0.041 × 0.022
2θ Range for data collection (°)	4.82 to 54.404	4.244 to 50.054
Limiting indices	$-27 \leq h \geq 26$, $-18 \leq k \geq 18$, $-25 \leq l \geq 25$	$-11 \leq h \geq 11$, $-16 \leq k \geq 16$, $-20 \leq l \geq 20$
Reflections collected	72174	55640
Independent reflections [R_{int}]	13267 [R_{int} = 0.0680]	7628 [R_{int} = 0.1255]
Data / restraints / parameters	13267/0/649	7628 /0/528
Goodness-of-fit on F^2	1.044	1.017
Final *R* indices [I>2σ(I)]	R_1 = 0.0477, wR_2 = 0.1096	R_1 = 0.0520, wR_2 = 0.1152
R indices (all data)	R_1 = 0.0889, wR_2 = 0.1251	R_1 = 0.1118, wR_2 = 0.1422
Largest diff. peak and hole (e. Å^{-3})	0.92/–0.94	1.81/–0.74

Table S2: Selected bond distances (Å) and angles (º) **2-3**

**Compound**	**Bond distances**		**Selected angles**	
**2**	Ru(2)–Ru(3)	2.6921(5)	C(8)–Ru(3)–Ru(2)	55.17(13)
	Ru(2)–C(8)	2.249(5)	C(38)–Ru(3)–C(37)	35.61(14)
	Ru(3)–C(8)	1.953(5)	P(2)–Ru(1)–P(1)	83.38(4)
	Ru(1)–C(9)	2.146(4)	Ru(2)–P(2)–Ru(1)	125.89(5)
	Ru(2)–C(37)	2.137(4)	C(37)–Ru(3)–Ru(2)	50.12(10)
	Ru(2)–C(40)	2.122(4)	Ru(3)–C(8)–Ru(2)	79.35(17)
	Ru(3)–C(37)	2.274(4)	C(38)–Ru(3)–Ru(2)	73.99(10)
	Ru(3)–C(38)	2.336(4)		
	Ru(3)–C(39)	2.354(4)		
	Ru(3)–C(40)	2.302(4)		
	Ru(2)–C(37)	2.137(4)		
	Ru(1)–P(1)	2.3907(11)		
	Ru(1)–P(2)	2.4230 (11)		
	Ru(2)–P(2)	2.3585 (11)		
	Ru(1)–C(9)	2.146(4)		
	C(9)–C(10)	1.336(6)		
**3**	Ru(2)–Ru(3)	2.7408(8)	C(31)–Ru(2)–Ru(3)	73.00(18)
	Ru(3)–C(30)	2.102(7)	C(31)–C(30)–Ru(2)	71.3(4)
	Ru(3)–C(33)	2.080(7)	P(2)–Ru(1)–P(1)	84.77(6)
	Ru(1)–C(9)	2.136(8)	Ru(1)–P(2)–Ru(2)	123.08(8)
	Ru(2)–C(30)	2.271(7)	C(9)–Ru(1)–P(2)	82.4(2)
	Ru(2)–C(31)	2.252(7)	C(9)–Ru(1)–P(1)	92.50(19)
	Ru(2)–C(33)	2.399(7)	Ru(2)–C(30)–Ru(3)	77.5(2)
	Ru(2)–C(32)	2.335(6)	C(33)–Ru(2)–Ru(3)	47.17 (17)
	Ru(3)–C(30)	2.102(7)		
	C(22)–C(23)	1.395(10)		
	C(32)–C(33)	1.442(9)		
	C(10)–C(11)	1.332(10)		
	Ru(1)–P(1)	2.3752(19)		
	Ru(1)–P(2)	2.3870(19)		
	Ru(2)–P(2)	2.3784(19)		

Table S3 Fractional Atomic Coordinates (×10[4]) and Equivalent Isotropic Displacement Parameters (Å[2]×10[3]) for **2**.

Atom	*x*	*y*	*z*	U(eq)
Ru1	6393.3(2)	8076.5(2)	2816.6(2)	29.38(10)
Ru2	7680.7(2)	7010.2(2)	4702.2(2)	32.78(10)
Ru3	8454.4(2)	5689.8(3)	5446.2(2)	35.59(11)
P1	6763.6(5)	7457.1(8)	1887.2(5)	31.1(3)
P2	7449.4(5)	7566.4(7)	3543.6(5)	28.7(2)
C1	5687(2)	8730(4)	2167(2)	44.5(12)
C2	5909(2)	6959(3)	2891(2)	39.1(11)
C3	6254(2)	8620(3)	3642(2)	39.6(11)
C4	6773(3)	6946(3)	4674(2)	42.6(11)
C5	7790(3)	8220(4)	5075(2)	48.6(13)
C6	8257(3)	4596(4)	5811(3)	50.5(13)
C7	9219(3)	5754(4)	6200(3)	53.2(13)
C8	7909(2)	6505(4)	5814(2)	48.3(12)
O1	5278.4(19)	9101(3)	1779(2)	75.6(12)
O2	5664.5(18)	6316(3)	2992(2)	65.4(11)
O3	6184.1(18)	8954(2)	4131.4(18)	59.1(10)
O4	6244.8(19)	6906(3)	4665(2)	68.1(11)
O5	7859(2)	8944(3)	5308(2)	80.5(13)
O6	8127(2)	3930(3)	6030(2)	82.2(13)
O7	9689(2)	5793(3)	6647(2)	88.5(14)

Table S3 Fractional Atomic Coordinates (×10^4) and Equivalent Isotropic Displacement Parameters (Å^2×10^3) for **2**.

Atom	*x*	*y*	*z*	U(eq)
O8	7717(2)	6703(3)	6291.1(18)	71.6(12)
C9	7142(2)	9099(3)	2983(2)	34.2(10)
C10	7691(2)	8728(3)	3391(2)	31.9(10)
C11	7788(2)	6844(3)	2976(2)	34.0(10)
C12	7635(2)	7239(3)	2236(2)	33.1(10)
C13	6375(2)	6366(3)	1555(2)	38.4(11)
C14	6707(3)	5561(3)	1585(2)	46.4(12)
C15	6368(3)	4761(4)	1328(3)	63.6(16)
C16	5707(3)	4785(4)	1030(3)	68.5(17)
C17	5371(3)	5586(4)	991(3)	71.4(18)
C18	5704(3)	6385(4)	1249(3)	52.4(13)
C19	6711(2)	8094(3)	1084(2)	35.6(10)
C20	6829(2)	7642(4)	520(2)	49.4(13)
C21	6856(3)	8118(4)	-69(3)	62.7(16)
C22	6757(3)	9040(4)	-102(3)	64.6(16)
C23	6641(3)	9504(4)	451(3)	59.6(15)
C24	6616(2)	9030(3)	1044(2)	44.6(12)
C25	8347(2)	9155(3)	3661(2)	40.5(11)
C26	8426(3)	9988(4)	3995(3)	59.9(15)
C27	9025(3)	10404(5)	4220(4)	83(2)

Table S3 Fractional Atomic Coordinates (×10^4) and Equivalent Isotropic Displacement Parameters (Å^2×10^3) for **2**.

Atom	*x*	*y*	*z*	U(eq)
C28	9558(3)	9981(5)	4091(4)	81(2)
C29	9490(3)	9166(5)	3776(3)	73.6(18)
C30	8892(2)	8743(4)	3564(3)	59.1(15)
C31	7079(2)	10055(3)	2718(2)	37.2(10)
C32	6597(3)	10619(3)	2824(3)	57.9(14)
C33	6549(3)	11520(4)	2584(4)	83(2)
C34	6965(4)	11841(4)	2225(4)	89(2)
C35	7432(3)	11269(4)	2100(3)	67.2(16)
C36	7493(3)	10399(3)	2351(3)	50.7(13)
C37	7718(2)	5629(3)	4364(2)	32.5(10)
C38	8315(2)	5340(3)	4266(2)	30.8(9)
C39	8845(2)	6005(3)	4479(2)	32.0(10)
C40	8680(2)	6839(3)	4761(2)	34.9(10)
C41	9199(2)	7457(3)	5024(2)	46.4(12)
C42	9816(3)	7288(4)	5003(3)	57.8(14)
C43	9968(2)	6467(4)	4709(3)	53.1(13)
C44	9497(2)	5836(3)	4465(2)	40.4(11)
C45	8406(2)	4449(3)	3940(2)	37.2(10)
C46	8615(2)	4450(4)	3336(2)	50.5(13)
C47	8682(3)	3648(4)	3005(3)	71.0(18)

Table S3 Fractional Atomic Coordinates (×10^4) and Equivalent Isotropic Displacement Parameters (Å^2×10^3) for **2**.

Atom	*x*	*y*	*z*	U(eq)
C48	8525(3)	2829(4)	3268(4)	76.9(19)
C49	8312(3)	2809(4)	3852(3)	68.9(17)
C50	8260(2)	3626(3)	4183(3)	49.0(12)
C51	7127(2)	5040(3)	4134(2)	34.0(10)
C52	6901(2)	4727(3)	3446(2)	42.2(11)
C53	6338(3)	4213(4)	3223(3)	56.1(14)
C54	5996(3)	3998(4)	3691(3)	63.1(15)
C55	6204(3)	4291(4)	4370(3)	55.9(14)
C56	6760(2)	4811(3)	4591(2)	43.7(12)
C57	8508(4)	9070(7)	981(5)	138(3)
Cl1	8703.0(14)	8093.0(18)	657.7(16)	146.0(10)
Cl2	8901.9(14)	9228(2)	1844.0(16)	167.1(12)

Table S4 Anisotropic Displacement Parameters (Å^2×10^3) for **2**.

Atom	U_{11}	U_{22}	U_{33}	U_{23}	U_{13}	U_{12}
Ru1	26.46(19)	32.16(19)	29.53(18)	0.10(15)	8.12(14)	1.01(15)
Ru2	38.1(2)	33.1(2)	27.61(18)	0.05(15)	10.15(15)	3.32(16)
Ru3	40.8(2)	38.7(2)	26.21(18)	4.87(16)	7.97(15)	1.14(17)
P1	33.1(6)	33.0(6)	27.3(6)	-0.8(5)	9.0(5)	-1.2(5)
P2	29.6(6)	28.6(6)	27.8(5)	1.2(5)	8.0(5)	1.7(5)

Table S4 Anisotropic Displacement Parameters (Å^2×10^3) for **2**.

Atom	U_{11}	U_{22}	U_{33}	U_{23}	U_{13}	U_{12}
C1	35(3)	58(3)	42(3)	1(2)	13(2)	-1(2)
C2	32(3)	49(3)	37(2)	0(2)	11(2)	-1(2)
C3	35(3)	41(3)	45(3)	2(2)	13(2)	1(2)
C4	48(3)	43(3)	39(3)	3(2)	15(2)	9(2)
C5	61(3)	51(3)	35(3)	-3(2)	14(2)	3(3)
C6	63(4)	52(3)	42(3)	12(2)	22(3)	5(3)
C7	51(3)	72(4)	35(3)	8(3)	9(3)	-2(3)
C8	50(3)	59(3)	32(3)	6(2)	7(2)	2(3)
O1	49(2)	100(3)	71(3)	30(2)	7(2)	24(2)
O2	56(2)	63(3)	76(3)	8(2)	18(2)	-20(2)
O3	71(3)	61(2)	51(2)	-10.3(19)	26(2)	10(2)
O4	49(2)	70(3)	97(3)	10(2)	40(2)	7(2)
O5	116(4)	47(2)	79(3)	-25(2)	27(3)	-1(2)
O6	112(4)	63(3)	82(3)	30(2)	44(3)	-5(3)
O7	63(3)	126(4)	57(3)	9(3)	-13(2)	1(3)
O8	96(3)	89(3)	36(2)	6(2)	28(2)	31(2)
C9	34(2)	35(2)	34(2)	-2(2)	11(2)	-1(2)
C10	37(3)	27(2)	31(2)	0.4(18)	8.2(19)	-2.0(19)
C11	35(2)	35(2)	31(2)	-0.2(19)	8.7(19)	8(2)
C12	37(3)	38(2)	28(2)	0.4(19)	14.9(19)	1(2)
C13	49(3)	38(3)	32(2)	-3(2)	17(2)	-9(2)

Table S4 Anisotropic Displacement Parameters (Å^2×10^3) for **2**.

Atom	U_{11}	U_{22}	U_{33}	U_{23}	U_{13}	U_{12}
C14	63(3)	36(3)	43(3)	-1(2)	20(2)	-1(2)
C15	96(5)	38(3)	65(4)	-7(3)	35(4)	-4(3)
C16	79(5)	58(4)	77(4)	-33(3)	37(4)	-36(3)
C17	61(4)	76(4)	82(4)	-31(4)	29(3)	-32(3)
C18	50(3)	53(3)	53(3)	-11(3)	14(3)	-10(3)
C19	35(2)	39(3)	29(2)	4(2)	5.0(19)	-3(2)
C20	62(3)	55(3)	32(2)	4(2)	14(2)	9(3)
C21	83(4)	79(4)	31(3)	2(3)	24(3)	4(3)
C22	87(4)	70(4)	39(3)	18(3)	21(3)	6(3)
C23	77(4)	48(3)	51(3)	11(3)	14(3)	-1(3)
C24	53(3)	44(3)	37(3)	3(2)	13(2)	1(2)
C25	37(3)	39(3)	43(3)	4(2)	7(2)	-4(2)
C26	40(3)	57(3)	77(4)	-20(3)	7(3)	-9(3)
C27	70(5)	69(4)	97(5)	-18(4)	3(4)	-25(4)
C28	41(4)	92(5)	94(5)	16(4)	-6(3)	-29(4)
C29	39(3)	84(5)	98(5)	-2(4)	19(3)	-1(3)
C30	38(3)	55(3)	82(4)	-6(3)	14(3)	-2(3)
C31	41(3)	29(2)	37(2)	2(2)	4(2)	-1(2)
C32	59(4)	42(3)	74(4)	5(3)	20(3)	7(3)
C33	79(5)	48(4)	124(6)	5(4)	30(4)	23(3)
C34	90(5)	51(4)	115(6)	36(4)	9(5)	-4(4)

Table S4 Anisotropic Displacement Parameters (Å^2×10^3) for **2**.

Atom	U_{11}	U_{22}	U_{33}	U_{23}	U_{13}	U_{12}
C35	75(4)	54(4)	71(4)	15(3)	18(3)	-12(3)
C36	59(3)	41(3)	53(3)	4(2)	17(3)	-8(3)
C37	36(3)	32(2)	28(2)	4.6(19)	7.8(19)	-2.0(19)
C38	36(2)	31(2)	25(2)	2.2(18)	8.7(18)	0.2(19)
C39	35(2)	38(2)	22(2)	0.8(18)	6.7(18)	-5(2)
C40	38(3)	37(2)	27(2)	4.2(19)	4.4(19)	-3(2)
C41	50(3)	39(3)	44(3)	-4(2)	4(2)	-6(2)
C42	44(3)	55(3)	68(4)	3(3)	7(3)	-21(3)
C43	33(3)	63(4)	60(3)	7(3)	7(2)	-1(3)
C44	39(3)	43(3)	38(3)	3(2)	9(2)	4(2)
C45	35(3)	34(2)	40(3)	-5(2)	7(2)	1(2)
C46	54(3)	55(3)	43(3)	-8(3)	16(2)	-1(3)
C47	75(4)	83(5)	61(4)	-32(3)	30(3)	-4(4)
C48	77(5)	56(4)	93(5)	-41(4)	18(4)	0(3)
C49	76(4)	37(3)	89(5)	-11(3)	17(4)	-4(3)
C50	49(3)	41(3)	54(3)	-1(2)	10(2)	2(2)
C51	36(3)	25(2)	39(2)	4.1(19)	8(2)	-1.9(19)
C52	45(3)	42(3)	40(3)	-1(2)	13(2)	-9(2)
C53	53(3)	56(3)	50(3)	-12(3)	0(3)	-10(3)
C54	44(3)	61(4)	81(4)	3(3)	14(3)	-18(3)
C55	44(3)	60(3)	68(4)	15(3)	24(3)	-5(3)

Table S4 Anisotropic Displacement Parameters (Å^2×10^3) for **2**.

Atom	U_{11}	U_{22}	U_{33}	U_{23}	U_{13}	U_{12}
C56	45(3)	46(3)	44(3)	7(2)	19(2)	-2(2)
C57	113(7)	165(9)	122(7)	-12(7)	14(6)	51(7)
Cl1	144(2)	121(2)	171(3)	-33.4(17)	41.5(19)	-8.2(16)
Cl2	124(2)	232(3)	155(2)	-68(2)	55.7(19)	1(2)

Table S5 Bond Lengths for **2**

Atom	Atom	Length/Å	Atom	Atom	Length/Å
Ru1	P1	2.3907(11)	C19	C20	1.388(6)
Ru1	P2	2.4230(11)	C19	C24	1.382(6)
Ru1	C1	1.933(5)	C20	C21	1.379(7)
Ru1	C2	1.964(5)	C21	C22	1.362(8)
Ru1	C3	1.924(5)	C22	C23	1.376(7)
Ru1	C9	2.146(4)	C23	C24	1.383(6)
Ru2	Ru3	2.6921(5)	C25	C26	1.375(7)
Ru2	P2	2.3585(11)	C25	C30	1.375(7)
Ru2	C4	1.930(5)	C26	C27	1.372(7)
Ru2	C5	1.906(5)	C27	C28	1.385(9)
Ru2	C8	2.249(5)	C28	C29	1.334(8)
Ru2	C37	2.137(4)	C29	C30	1.373(7)
Ru2	C40	2.122(4)	C31	C32	1.382(6)
Ru3	C6	1.855(5)	C31	C36	1.396(6)

Table S5 Bond Lengths for **2**

Atom	Atom	Length/Å	Atom	Atom	Length/Å
Ru3	C7	1.876(5)	C32	C33	1.395(7)
Ru3	C8	1.953(5)	C33	C34	1.376(9)
Ru3	C37	2.274(4)	C34	C35	1.380(9)
Ru3	C38	2.336(4)	C35	C36	1.359(7)
Ru3	C39	2.354(4)	C37	C38	1.411(6)
Ru3	C40	2.302(4)	C37	C51	1.489(6)
P1	C12	1.820(4)	C38	C39	1.462(6)
P1	C13	1.832(4)	C38	C45	1.495(6)
P1	C19	1.825(4)	C39	C40	1.429(6)
P2	C10	1.827(4)	C39	C44	1.425(6)
P2	C11	1.842(4)	C40	C41	1.412(6)
C1	O1	1.126(5)	C41	C42	1.354(7)
C2	O2	1.122(5)	C42	C43	1.415(7)
C3	O3	1.136(5)	C43	C44	1.349(6)
C4	O4	1.127(6)	C45	C46	1.398(6)
C5	O5	1.148(6)	C45	C50	1.366(6)
C6	O6	1.135(6)	C46	C47	1.373(7)
C7	O7	1.136(6)	C47	C48	1.386(8)
C8	O8	1.174(5)	C48	C49	1.366(8)
C9	C10	1.336(6)	C49	C50	1.385(7)
C9	C31	1.486(6)	C51	C52	1.392(6)

Table S5 Bond Lengths for **2**

Atom	Atom	Length/Å	Atom	Atom	Length/Å
C10	C25	1.488(6)	C51	C56	1.403(6)
C11	C12	1.526(5)	C52	C53	1.381(6)
C13	C14	1.368(6)	C53	C54	1.377(7)
C13	C18	1.389(7)	C54	C55	1.363(7)
C14	C15	1.393(7)	C55	C56	1.373(7)
C15	C16	1.367(8)	C57	Cl1	1.668(9)
C16	C17	1.365(8)	C57	Cl2	1.696(9)
C17	C18	1.387(7)			

Table S6 Bond Angles for **2**.

Atom	Atom	Atom	Angle/˚	Atom	Atom	Atom	Angle/˚
P1	Ru1	P2	83.38(4)	C10	C9	Ru1	107.3(3)
C1	Ru1	P1	92.29(14)	C10	C9	C31	124.9(4)
C1	Ru1	P2	164.91(14)	C31	C9	Ru1	127.8(3)
C1	Ru1	C2	97.7(2)	C9	C10	P2	103.7(3)
C1	Ru1	C9	99.38(19)	C9	C10	C25	128.5(4)
C2	Ru1	P1	92.47(13)	C25	C10	P2	127.8(3)
C2	Ru1	P2	96.92(13)	C12	C11	P2	111.2(3)
C2	Ru1	C9	162.77(17)	C11	C12	P1	110.2(3)
C3	Ru1	P1	169.96(14)	C14	C13	P1	123.9(4)
C3	Ru1	P2	87.99(13)	C14	C13	C18	119.6(4)

Table S6 Bond Angles for **2**.

Atom	Atom	Atom	Angle/˚	Atom	Atom	Atom	Angle/˚
C3	Ru1	C1	94.76(19)	C18	C13	P1	116.5(4)
C3	Ru1	C2	93.66(19)	C13	C14	C15	119.9(5)
C3	Ru1	C9	82.75(17)	C16	C15	C14	120.1(5)
C9	Ru1	P1	89.06(11)	C17	C16	C15	120.6(5)
C9	Ru1	P2	66.20(12)	C16	C17	C18	119.7(6)
P2	Ru2	Ru3	135.01(3)	C17	C18	C13	120.1(5)
C4	Ru2	Ru3	115.82(14)	C20	C19	P1	119.0(4)
C4	Ru2	P2	93.59(14)	C24	C19	P1	121.8(3)
C4	Ru2	C8	86.78(19)	C24	C19	C20	118.9(4)
C4	Ru2	C37	94.21(18)	C21	C20	C19	120.6(5)
C4	Ru2	C40	170.32(18)	C22	C21	C20	119.8(5)
C5	Ru2	Ru3	117.67(15)	C21	C22	C23	120.7(5)
C5	Ru2	P2	91.61(14)	C22	C23	C24	119.8(5)
C5	Ru2	C4	93.8(2)	C19	C24	C23	120.3(5)
C5	Ru2	C8	87.38(19)	C26	C25	C10	121.2(4)
C5	Ru2	C37	171.00(19)	C30	C25	C10	121.1(4)
C5	Ru2	C40	94.6(2)	C30	C25	C26	117.7(5)
C8	Ru2	Ru3	45.48(13)	C27	C26	C25	121.5(6)
C8	Ru2	P2	178.94(14)	C26	C27	C28	119.0(6)
C37	Ru2	Ru3	54.73(11)	C29	C28	C27	120.1(6)
C37	Ru2	P2	91.98(11)	C28	C29	C30	120.8(6)

Table S6 Bond Angles for **2**.

Atom	Atom	Atom	Angle/°	Atom	Atom	Atom	Angle/°
C37	Ru2	C8	88.98(17)	C29	C30	C25	120.8(5)
C40	Ru2	Ru3	55.61(11)	C32	C31	C9	120.2(4)
C40	Ru2	P2	91.04(11)	C32	C31	C36	118.6(4)
C40	Ru2	C8	88.75(17)	C36	C31	C9	121.2(4)
C40	Ru2	C37	77.12(16)	C31	C32	C33	119.8(5)
C6	Ru3	Ru2	131.30(16)	C34	C33	C32	120.3(6)
C6	Ru3	C7	88.6(2)	C33	C34	C35	119.8(6)
C6	Ru3	C8	98.7(2)	C36	C35	C34	120.0(6)
C6	Ru3	C37	99.38(19)	C35	C36	C31	121.4(5)
C6	Ru3	C38	103.14(18)	Ru2	C37	Ru3	75.15(13)
C6	Ru3	C39	131.57(18)	C38	C37	Ru2	116.3(3)
C6	Ru3	C40	166.39(19)	C38	C37	Ru3	74.6(2)
C7	Ru3	Ru2	131.29(17)	C38	C37	C51	120.5(4)
C7	Ru3	C8	98.5(2)	C51	C37	Ru2	122.5(3)
C7	Ru3	C37	164.89(18)	C51	C37	Ru3	127.5(3)
C7	Ru3	C38	130.06(19)	C37	C38	Ru3	69.8(2)
C7	Ru3	C39	102.11(18)	C37	C38	C39	114.8(4)
C7	Ru3	C40	98.85(19)	C37	C38	C45	124.0(4)
C8	Ru3	Ru2	55.17(13)	C39	C38	Ru3	72.5(2)
C8	Ru3	C37	93.03(17)	C39	C38	C45	121.2(4)
C8	Ru3	C38	126.39(17)	C45	C38	Ru3	129.3(3)

Table S6 Bond Angles for **2**.

Atom	Atom	Atom	Angle/˚	Atom	Atom	Atom	Angle/˚
C8	Ru3	C39	125.37(18)	C38	C39	Ru3	71.2(2)
C8	Ru3	C40	91.53(18)	C40	C39	Ru3	70.1(2)
C37	Ru3	Ru2	50.12(10)	C40	C39	C38	114.9(4)
C37	Ru3	C38	35.61(14)	C44	C39	Ru3	124.3(3)
C37	Ru3	C39	63.03(14)	C44	C39	C38	124.2(4)
C37	Ru3	C40	70.95(14)	C44	C39	C40	120.7(4)
C38	Ru3	Ru2	73.99(10)	Ru2	C40	Ru3	74.84(14)
C38	Ru3	C39	36.31(14)	C39	C40	Ru2	116.1(3)
C39	Ru3	Ru2	73.59(10)	C39	C40	Ru3	74.2(2)
C40	Ru3	Ru2	49.55(11)	C41	C40	Ru2	128.2(3)
C40	Ru3	C38	63.37(14)	C41	C40	Ru3	121.7(3)
C40	Ru3	C39	35.71(14)	C41	C40	C39	115.8(4)
C12	P1	Ru1	107.65(13)	C42	C41	C40	122.8(5)
C12	P1	C13	107.1(2)	C41	C42	C43	120.7(5)
C12	P1	C19	102.78(19)	C44	C43	C42	119.6(5)
C13	P1	Ru1	113.03(14)	C43	C44	C39	120.5(4)
C19	P1	Ru1	122.22(15)	C46	C45	C38	119.1(4)
C19	P1	C13	102.8(2)	C50	C45	C38	122.8(4)
Ru2	P2	Ru1	125.89(5)	C50	C45	C46	118.0(4)
C10	P2	Ru1	82.80(14)	C47	C46	C45	121.0(5)
C10	P2	Ru2	119.57(14)	C46	C47	C48	119.1(5)

Table S6 Bond Angles for **2**.

Atom	Atom	Atom	Angle/°	Atom	Atom	Atom	Angle/°
C10	P2	C11	104.53(19)	C49	C48	C47	121.1(5)
C11	P2	Ru1	105.96(14)	C48	C49	C50	118.6(6)
C11	P2	Ru2	113.16(13)	C45	C50	C49	122.2(5)
O1	C1	Ru1	178.7(5)	C52	C51	C37	121.0(4)
O2	C2	Ru1	174.0(4)	C52	C51	C56	117.3(4)
O3	C3	Ru1	178.4(4)	C56	C51	C37	121.6(4)
O4	C4	Ru2	179.1(4)	C53	C52	C51	121.2(4)
O5	C5	Ru2	179.2(5)	C54	C53	C52	119.5(5)
O6	C6	Ru3	179.1(5)	C55	C54	C53	120.8(5)
O7	C7	Ru3	178.7(5)	C54	C55	C56	119.9(5)
Ru3	C8	Ru2	79.35(17)	C55	C56	C51	121.3(5)
O8	C8	Ru2	133.4(4)	Cl1	C57	Cl2	113.7(5)
O8	C8	Ru3	147.2(4)				

Table S7 Torsion Angles for **2**.

A	B	C	D	Angle/°	A	B	C	D	Angle/°
Ru1	P1	C12	C11	40.5(3)	C19	P1	C13	C14	108.3(4)
Ru1	P1	C13	C14	-118.0(4)	C19	P1	C13	C18	-70.7(4)
Ru1	P1	C13	C18	63.0(4)	C19	C20	C21	C22	-0.8(9)
Ru1	P1	C19	C20	-169.0(3)	C20	C19	C24	C23	-0.1(7)
Ru1	P1	C19	C24	17.1(5)	C20	C21	C22	C23	1.0(9)

Table S7 Torsion Angles for **2**.

A	B	C	D	Angle/°	A	B	C	D	Angle/°
Ru1	P2	C10	C9	-0.3(3)	C21	C22	C23	C24	-0.6(9)
Ru1	P2	C10	C25	179.6(4)	C22	C23	C24	C19	0.2(8)
Ru1	P2	C11	C12	39.1(3)	C24	C19	C20	C21	0.4(7)
Ru1	C9	C10	P2	0.4(3)	C25	C26	C27	C28	1.6(10)
Ru1	C9	C10	C25	-179.6(4)	C26	C25	C30	C29	-1.7(8)
Ru1	C9	C31	C32	51.5(6)	C26	C27	C28	C29	-2.4(10)
Ru1	C9	C31	C36	-127.6(4)	C27	C28	C29	C30	1.2(10)
Ru2	P2	C10	C9	-127.7(3)	C28	C29	C30	C25	0.9(10)
Ru2	P2	C10	C25	52.2(4)	C30	C25	C26	C27	0.5(8)
Ru2	P2	C11	C12	-179.1(3)	C31	C9	C10	P2	179.0(3)
Ru2	C37	C38	Ru3	64.4(2)	C31	C9	C10	C25	-0.9(7)
Ru2	C37	C38	C39	5.9(5)	C31	C32	C33	C34	2.0(10)
Ru2	C37	C38	C45	-171.3(3)	C32	C31	C36	C35	0.0(7)
Ru2	C37	C51	C52	115.4(4)	C32	C33	C34	C35	0.0(11)
Ru2	C37	C51	C56	-61.9(5)	C33	C34	C35	C36	-2.0(10)
Ru2	C40	C41	C42	-176.8(4)	C34	C35	C36	C31	1.9(9)
Ru3	C37	C38	C39	-58.5(3)	C36	C31	C32	C33	-2.0(8)
Ru3	C37	C38	C45	124.4(4)	C37	C38	C39	Ru3	57.0(3)
Ru3	C37	C51	C52	-148.5(3)	C37	C38	C39	C40	0.6(5)
Ru3	C37	C51	C56	34.2(6)	C37	C38	C39	C44	176.2(4)
Ru3	C38	C39	C40	-56.3(3)	C37	C38	C45	C46	121.0(5)

Table S7 Torsion Angles for **2**.

A	B	C	D	Angle/°	A	B	C	D	Angle/°
Ru3	C38	C39	C44	119.2(4)	C37	C38	C45	C50	-55.2(6)
Ru3	C38	C45	C46	-148.3(4)	C37	C51	C52	C53	-177.3(4)
Ru3	C38	C45	C50	35.5(6)	C37	C51	C56	C55	177.9(4)
Ru3	C39	C40	Ru2	-63.8(2)	C38	C37	C51	C52	-54.9(6)
Ru3	C39	C40	C41	118.0(4)	C38	C37	C51	C56	127.8(4)
Ru3	C39	C44	C43	-86.6(5)	C38	C39	C40	Ru2	-6.9(5)
Ru3	C40	C41	C42	87.8(5)	C38	C39	C40	Ru3	56.9(3)
P1	C13	C14	C15	179.1(4)	C38	C39	C40	C41	174.9(4)
P1	C13	C18	C17	-179.4(4)	C38	C39	C44	C43	-176.1(4)
P1	C19	C20	C21	-173.7(4)	C38	C45	C46	C47	-177.6(5)
P1	C19	C24	C23	173.8(4)	C38	C45	C50	C49	176.3(5)
P2	C10	C25	C26	-129.3(4)	C39	C38	C45	C46	-56.0(6)
P2	C10	C25	C30	53.0(6)	C39	C38	C45	C50	127.8(5)
P2	C11	C12	P1	-52.5(4)	C39	C40	C41	C42	1.2(7)
C9	C10	C25	C26	50.6(7)	C40	C39	C44	C43	-0.8(6)
C9	C10	C25	C30	-127.0(5)	C40	C41	C42	C43	0.1(8)
C9	C31	C32	C33	178.9(5)	C41	C42	C43	C44	-1.8(8)
C9	C31	C36	C35	179.2(5)	C42	C43	C44	C39	2.1(7)
C10	P2	C11	C12	-47.4(3)	C44	C39	C40	Ru2	177.4(3)
C10	C9	C31	C32	-126.9(5)	C44	C39	C40	Ru3	-118.8(4)
C10	C9	C31	C36	54.0(6)	C44	C39	C40	C41	-0.9(6)

Table S7 Torsion Angles for **2**.

A	B	C	D	Angle/˚	A	B	C	D	Angle/˚
C10	C25	C26	C27	-177.3(5)	C45	C38	C39	Ru3	-125.8(4)
C10	C25	C30	C29	176.0(5)	C45	C38	C39	C40	177.9(4)
C11	P2	C10	C9	104.4(3)	C45	C38	C39	C44	-6.6(6)
C11	P2	C10	C25	-75.7(4)	C45	C46	C47	C48	1.5(8)
C12	P1	C13	C14	0.4(4)	C46	C45	C50	C49	0.0(7)
C12	P1	C13	C18	-178.6(3)	C46	C47	C48	C49	-0.6(9)
C12	P1	C19	C20	70.3(4)	C47	C48	C49	C50	-0.5(9)
C12	P1	C19	C24	-103.6(4)	C48	C49	C50	C45	0.8(8)
C13	P1	C12	C11	-81.4(3)	C50	C45	C46	C47	-1.2(7)
C13	P1	C19	C20	-40.9(4)	C51	C37	C38	Ru3	-124.8(4)
C13	P1	C19	C24	145.2(4)	C51	C37	C38	C39	176.8(3)
C13	C14	C15	C16	1.6(8)	C51	C37	C38	C45	-0.4(6)
C14	C13	C18	C17	1.6(7)	C51	C52	C53	C54	-0.7(8)
C14	C15	C16	C17	-1.1(9)	C52	C51	C56	C55	0.5(7)
C15	C16	C17	C18	0.7(9)	C52	C53	C54	C55	0.5(8)
C16	C17	C18	C13	-1.0(8)	C53	C54	C55	C56	0.2(8)
C18	C13	C14	C15	-1.9(7)	C54	C55	C56	C51	-0.7(8)
C19	P1	C12	C11	170.7(3)	C56	C51	C52	C53	0.2(7)

Table S8 Hydrogen Atom Coordinates (Å×10^4) and Isotropic Displacement Parameters (Å^2×10^3) for **2**

Atom	*x*	*y*	*z*	U(eq)
H11A	8262.36	6797.21	3178.13	41
H11B	7604.26	6227.38	2952.74	41
H12A	7773.16	6807.71	1930.18	40
H12B	7876.76	7810.77	2247.41	40
H14	7162.43	5546.3	1779.39	56
H15	6593.74	4204.29	1359.08	76
H16	5482.16	4244.98	849.77	82
H17	4915.83	5596.12	789.78	86
H18	5474.83	6939	1216.53	63
H20	6891.02	7005.27	539.32	59
H21	6941.71	7808.23	-446.51	75
H22	6769.48	9361.51	-507.22	78
H23	6577.43	10140.11	425.21	72
H24	6535.25	9345.98	1422.05	54
H26	8061.19	10280.45	4071.04	72
H27	9073.2	10966.6	4457.35	100
H28	9968.83	10267.81	4226.12	97
H29	9856.09	8878.24	3699.07	88
H30	8855.33	8165.76	3348.81	71
H32	6303.61	10396.1	3056.42	69

Table S8 Hydrogen Atom Coordinates (Å×10^4) and Isotropic Displacement Parameters (Å^2×10^3) for **2**

Atom	*x*	*y*	*z*	U(eq)
H33	6230.5	11909.5	2668.36	100
H34	6931.17	12448.61	2063.92	107
H35	7708.52	11480.68	1842.49	81
H36	7822.16	10020.55	2276.29	61
H41	9114.03	8009.92	5222.06	56
H42	10145.54	7721.09	5185.77	69
H43	10393.74	6361.02	4684.79	64
H44	9599.91	5280.6	4283.28	48
H46	8710.85	5009	3153.56	61
H47	8832.66	3654.33	2605.14	85
H48	8566.57	2278.83	3040.16	92
H49	8203.18	2252.13	4025.29	83
H50	8118.55	3615	4588.13	59
H52	7136.42	4867.93	3127	51
H53	6189.05	4011.2	2755.4	67
H54	5614.37	3643.68	3541.12	76
H55	5967.49	4138.56	4684.93	67
H56	6897.65	5016.89	5058.66	52
H57A	8036.83	9078.06	916.75	165
H57B	8611.31	9580.96	713.15	165

Table S9 Fractional Atomic Coordinates (×10^4) and Equivalent Isotropic Displacement Parameters (Å^2×10^3) for **3**

**Atom**	***x***	***y***	***z***	**U(eq)**
Ru1	3835.3(6)	2748.6(4)	2182.4(3)	23.88(18)
Ru2	4413.1(6)	5582.5(4)	2516.2(3)	23.80(18)
Ru3	2758.0(6)	7267.8(5)	1720.5(4)	26.85(18)
P1	1396.2(19)	2797.7(15)	2622.5(11)	24.5(4)
P2	3257.4(18)	4214.5(14)	2907.2(11)	22.9(4)
C1	3342(7)	3648(6)	1182(4)	25.5(17)
C2	4289(8)	1429(7)	1739(5)	45(2)
C3	5821(8)	2760(5)	1970(4)	28.2(17)
C4	5036(7)	5165(5)	1503(5)	29.7(18)
C5	5879(8)	4725(6)	3028(5)	31.8(19)
C6	3327(8)	7195(6)	581(5)	34.1(19)
C7	927(9)	7068(6)	1731(5)	41(2)
C8	2260(8)	8725(7)	1684(5)	37(2)
O1	3007(6)	4179(5)	648(3)	52.1(17)
O2	4533(7)	673(5)	1499(4)	70(2)
O3	6974(6)	2729(4)	1890(3)	47.4(16)
O4	5525(5)	4906(4)	879(3)	41.7(14)
O5	6808(6)	4217(5)	3327(4)	51.7(16)
O6	3652(7)	7179(5)	-91(4)	59.9(18)
O7	-134(6)	6945(5)	1724(4)	63.1(19)

Table S9 Fractional Atomic Coordinates (×10^4) and Equivalent Isotropic Displacement Parameters (Å^2×10^3) for **3**

**Atom**	***x***	***y***	***z***	**U(eq)**
O8	2044(6)	9612(5)	1701(4)	61.7(18)
O9	5013(6)	989(4)	3326(3)	43.4(14)
C9	4362(8)	1902(6)	3287(4)	31.9(18)
C10	4027(7)	2492(6)	3986(4)	30.7(18)
C11	3526(7)	3531(6)	3899(4)	26.1(17)
C12	3238(8)	4207(6)	4549(4)	32.0(18)
C13	1953(9)	4149(7)	5184(5)	46(2)
C14	4334(8)	1822(6)	4750(4)	37(2)
C15	3287(9)	1162(6)	5113(5)	47(2)
C16	1304(7)	4666(5)	3096(4)	26.5(17)
C17	604(7)	3778(5)	3319(4)	25.1(17)
C18	433(7)	3215(6)	1795(4)	26.9(17)
C19	-431(7)	4199(6)	1660(5)	37(2)
C20	-1023(8)	4507(7)	988(5)	45(2)
C21	-784(8)	3811(7)	443(5)	45(2)
C22	84(9)	2818(7)	580(5)	48(2)
C23	693(8)	2511(6)	1261(5)	35.4(19)
C24	809(7)	1660(5)	3141(4)	24.8(17)
C25	-615(8)	1718(6)	3310(5)	46(2)
C26	-1102(10)	868(8)	3713(6)	64(3)

Table S9 Fractional Atomic Coordinates (×10^4) and Equivalent Isotropic Displacement Parameters (Å^2×10^3) for **3**

**Atom**	***x***	***y***	***z***	**U(eq)**
C27	-145(10)	-48(7)	3942(6)	57(3)
C28	1263(10)	-117(7)	3755(6)	54(3)
C29	1771(8)	719(6)	3356(5)	39(2)
C30	4826(7)	7120(5)	1870(4)	29.3(18)
C31	5020(7)	7010(6)	2655(4)	29.1(18)
C32	3745(7)	6985(5)	3267(4)	21.5(16)
C33	2506(8)	7043(5)	2962(4)	30.1(18)
C34	1231(8)	7052(6)	3539(5)	34.2(19)
C35	1187(8)	7047(6)	4338(5)	36(2)
C36	2396(10)	6995(6)	4619(5)	43(2)
C37	3650(9)	6959(6)	4095(5)	38(2)
C38	6360(8)	7005(6)	2896(5)	40(2)
C39	6366(10)	8105(7)	2968(6)	61(3)
C40	6023(7)	7225(6)	1168(4)	33.1(19)
C41	5855(9)	8352(6)	765(5)	53(3)
C42	8366(12)	52(9)	1805(7)	99(4)
C43	8525(13)	244(10)	938(8)	100(4)
C44	9869(14)	-123(9)	411(6)	99(4)

Table S10 Anisotropic Displacement Parameters (Å^2×10^3) for **3**

**Atom**	**U$_{11}$**	**U$_{22}$**	**U$_{33}$**	**U$_{23}$**	**U$_{13}$**	**U$_{12}$**
Ru1	23.6(3)	22.0(4)	25.3(3)	-3.5(3)	-3.2(3)	-5.9(3)
Ru2	24.5(3)	21.8(3)	25.7(4)	-1.1(3)	-5.4(3)	-7.8(3)
Ru3	29.5(3)	24.2(4)	25.8(4)	-1.0(3)	-6.7(3)	-5.7(3)
P1	24.9(10)	24.4(11)	25.0(11)	-2.8(9)	-3.6(8)	-8.7(8)
P2	21.2(10)	23.7(11)	23.4(10)	-2.4(8)	-2.7(8)	-7.0(8)
C1	19(4)	32(5)	27(4)	-9(4)	2(3)	-10(3)
C2	28(5)	45(6)	62(6)	-14(5)	-4(4)	-6(4)
C3	40(5)	19(4)	26(4)	-1(3)	-8(4)	-8(4)
C4	32(4)	22(4)	34(5)	2(4)	0(4)	-16(3)
C5	33(5)	25(5)	38(5)	-4(4)	-3(4)	-12(4)
C6	31(4)	37(5)	34(5)	-6(4)	-8(4)	-5(4)
C7	40(5)	41(5)	39(5)	3(4)	-14(4)	-6(4)
C8	32(5)	34(5)	42(5)	0(4)	-8(4)	-6(4)
O1	51(4)	70(5)	34(4)	15(3)	-10(3)	-25(3)
O2	71(5)	43(4)	100(6)	-42(4)	-9(4)	-8(4)
O3	23(3)	46(4)	65(4)	-1(3)	-1(3)	-6(3)
O4	47(3)	45(4)	29(3)	-10(3)	9(3)	-15(3)
O5	35(3)	54(4)	61(4)	6(3)	-23(3)	-3(3)
O6	70(5)	73(5)	34(4)	-12(3)	-11(3)	-11(4)
O7	49(4)	75(5)	73(5)	10(4)	-25(4)	-29(4)
O8	67(4)	37(4)	78(5)	-7(4)	-19(4)	-6(3)

Table S10 Anisotropic Displacement Parameters (Å^2×10^3) for **3**

**Atom**	**U_{11}**	**U_{22}**	**U_{33}**	**U_{23}**	**U_{13}**	**U_{12}**
O9	55(4)	20(3)	58(4)	-7(3)	-31(3)	4(3)
C9	30(4)	35(5)	30(5)	-3(4)	1(3)	-14(4)
C10	30(4)	34(5)	29(4)	0(4)	-8(3)	-10(4)
C11	24(4)	31(5)	26(4)	-5(3)	-4(3)	-11(3)
C12	41(5)	34(5)	22(4)	2(3)	-12(4)	-10(4)
C13	56(6)	48(6)	28(5)	-8(4)	1(4)	-10(4)
C14	46(5)	28(5)	39(5)	8(4)	-17(4)	-12(4)
C15	61(6)	41(5)	41(5)	9(4)	-11(4)	-25(5)
C16	25(4)	30(4)	23(4)	-2(3)	-4(3)	-7(3)
C17	19(4)	29(4)	28(4)	-13(3)	3(3)	-9(3)
C18	15(4)	34(5)	34(4)	-2(4)	-4(3)	-12(3)
C19	24(4)	44(5)	40(5)	-5(4)	-4(4)	-10(4)
C20	39(5)	32(5)	57(6)	13(5)	-17(4)	-4(4)
C21	42(5)	59(6)	35(5)	12(5)	-16(4)	-20(5)
C22	52(6)	59(7)	42(5)	-18(5)	-15(5)	-16(5)
C23	36(5)	32(5)	38(5)	-2(4)	-15(4)	-2(4)
C24	27(4)	22(4)	26(4)	-6(3)	4(3)	-12(3)
C25	32(5)	32(5)	65(6)	1(4)	1(4)	-9(4)
C26	48(6)	59(7)	85(8)	-14(6)	20(5)	-35(5)
C27	65(7)	28(5)	70(7)	-3(5)	10(5)	-22(5)
C28	56(6)	27(5)	74(7)	8(5)	-10(5)	-13(4)

Table S10 Anisotropic Displacement Parameters ($Å^2 \times 10^3$) for **3**

**Atom**	**U_{11}**	**U_{22}**	**U_{33}**	**U_{23}**	**U_{13}**	**U_{12}**
C29	39(5)	28(5)	51(5)	5(4)	-12(4)	-16(4)
C30	34(4)	18(4)	36(5)	-6(3)	-4(4)	-7(3)
C31	25(4)	28(4)	37(5)	-2(4)	-9(4)	-11(3)
C32	37(4)	13(4)	20(4)	-4(3)	-11(3)	-8(3)
C33	45(5)	18(4)	31(4)	1(3)	-15(4)	-11(4)
C34	37(5)	27(5)	37(5)	-6(4)	1(4)	-11(4)
C35	44(5)	27(5)	30(5)	-3(4)	5(4)	-5(4)
C36	72(6)	29(5)	30(5)	-8(4)	-4(5)	-19(4)
C37	59(6)	25(5)	32(5)	-4(4)	-10(4)	-15(4)
C38	37(5)	58(6)	34(5)	-4(4)	-8(4)	-24(4)
C39	78(7)	62(7)	69(7)	-1(5)	-29(6)	-50(6)
C40	32(4)	25(4)	44(5)	-5(4)	1(4)	-16(4)
C41	65(6)	39(5)	47(6)	-6(4)	16(5)	-23(5)
C42	83(9)	71(9)	112(11)	26(8)	-9(8)	-8(7)
C43	91(9)	90(10)	92(10)	0(8)	-21(8)	13(7)
C44	108(10)	70(8)	81(9)	-8(8)	4(9)	13(7)

Table S11 Bond Lengths for **3**

**Atom**	**Atom**	**Length/Å**	**Atom**	**Atom**	**Length/Å**
Ru1	P1	2.3752(19)	C10	C11	1.332(10)
Ru1	P2	2.3870(19)	C10	C14	1.500(10)

Table S11 Bond Lengths for **3**

Atom	Atom	Length/Å	Atom	Atom	Length/Å
Ru1	C1	1.998(8)	C11	C12	1.500(9)
Ru1	C2	1.952(9)	C12	C13	1.498(10)
Ru1	C3	1.947(8)	C14	C15	1.529(10)
Ru1	C9	2.136(8)	C16	C17	1.510(9)
Ru2	Ru3	2.7408(8)	C18	C19	1.365(10)
Ru2	P2	2.3784(19)	C18	C23	1.373(10)
Ru2	C4	1.850(8)	C19	C20	1.372(10)
Ru2	C5	1.877(8)	C20	C21	1.383(11)
Ru2	C30	2.271(7)	C21	C22	1.376(11)
Ru2	C31	2.252(7)	C22	C23	1.395(10)
Ru2	C32	2.335(6)	C24	C25	1.374(10)
Ru2	C33	2.399(7)	C24	C29	1.396(10)
Ru3	C6	1.934(8)	C25	C26	1.384(11)
Ru3	C7	1.934(9)	C26	C27	1.379(12)
Ru3	C8	1.869(9)	C27	C28	1.354(11)
Ru3	C30	2.102(7)	C28	C29	1.378(10)
Ru3	C33	2.080(7)	C30	C31	1.393(10)
P1	C17	1.828(7)	C30	C40	1.515(9)
P1	C18	1.829(7)	C31	C32	1.463(9)
P1	C24	1.814(7)	C31	C38	1.500(9)
P2	C11	1.833(7)	C32	C33	1.442(9)

Table S11 Bond Lengths for **3**

Atom	Atom	Length/Å	Atom	Atom	Length/Å
P2	C16	1.843(7)	C32	C37	1.408(9)
C1	O1	1.116(8)	C33	C34	1.426(10)
C2	O2	1.110(9)	C34	C35	1.374(10)
C3	O3	1.124(8)	C35	C36	1.392(11)
C4	O4	1.147(8)	C36	C37	1.363(10)
C5	O5	1.152(8)	C38	C39	1.513(11)
C6	O6	1.135(8)	C40	C41	1.529(10)
C7	O7	1.130(9)	C42	C43	1.455(14)
C8	O8	1.155(9)	C43	C44	1.446(15)
O9	C9	1.208(8)	C44	C44[1]	1.38(2)
C9	C10	1.487(10)			

[1]2-X,-Y,-Z

Table S12 Bond Angles for **3**

Atom	Atom	Atom	Angle/°	Atom	Atom	Atom	Angle/°
P1	Ru1	P2	84.77(6)	O1	C1	Ru1	176.4(7)
C1	Ru1	P1	88.28(19)	O2	C2	Ru1	178.8(9)
C1	Ru1	P2	93.0(2)	O3	C3	Ru1	175.8(7)
C1	Ru1	C9	175.2(3)	O4	C4	Ru2	174.7(6)
C2	Ru1	P1	93.0(2)	O5	C5	Ru2	177.8(7)
C2	Ru1	P2	171.8(3)	O6	C6	Ru3	178.2(7)

Table S12 Bond Angles for **3**

**Atom**	**Atom**	**Atom**	**Angle/°**	**Atom**	**Atom**	**Atom**	**Angle/°**
C2	Ru1	C1	94.8(3)	O7	C7	Ru3	178.8(8)
C2	Ru1	C9	89.8(3)	O8	C8	Ru3	173.9(7)
C3	Ru1	P1	171.9(2)	O9	C9	Ru1	120.8(6)
C3	Ru1	P2	88.8(2)	O9	C9	C10	120.8(7)
C3	Ru1	C1	97.0(3)	C10	C9	Ru1	118.1(5)
C3	Ru1	C2	92.6(3)	C9	C10	C14	114.4(7)
C3	Ru1	C9	81.7(3)	C11	C10	C9	120.2(7)
C9	Ru1	P1	92.50(19)	C11	C10	C14	125.4(7)
C9	Ru1	P2	82.4(2)	C10	C11	P2	119.0(6)
P2	Ru2	Ru3	109.71(5)	C10	C11	C12	124.6(7)
P2	Ru2	C33	100.49(18)	C12	C11	P2	116.4(5)
C4	Ru2	Ru3	82.1(2)	C13	C12	C11	114.9(6)
C4	Ru2	P2	89.0(2)	C10	C14	C15	113.3(6)
C4	Ru2	C5	100.7(3)	C17	C16	P2	113.1(5)
C4	Ru2	C30	83.4(3)	C16	C17	P1	111.1(5)
C4	Ru2	C31	114.5(3)	C19	C18	P1	123.3(6)
C4	Ru2	C32	145.9(3)	C19	C18	C23	119.7(7)
C4	Ru2	C33	128.8(3)	C23	C18	P1	116.9(6)
C5	Ru2	Ru3	162.2(2)	C18	C19	C20	121.2(8)
C5	Ru2	P2	88.0(2)	C19	C20	C21	120.3(8)
C5	Ru2	C30	114.0(3)	C22	C21	C20	118.6(8)

Table S12 Bond Angles for **3**

Atom	Atom	Atom	Angle/°	Atom	Atom	Atom	Angle/°
C5	Ru2	C31	90.0(3)	C21	C22	C23	120.9(8)
C5	Ru2	C32	98.1(3)	C18	C23	C22	119.4(7)
C5	Ru2	C33	129.6(3)	C25	C24	P1	119.2(6)
C30	Ru2	Ru3	48.49(18)	C25	C24	C29	119.1(7)
C30	Ru2	P2	157.64(19)	C29	C24	P1	121.7(5)
C30	Ru2	C32	62.9(2)	C24	C25	C26	120.7(8)
C30	Ru2	C33	69.1(2)	C27	C26	C25	119.6(8)
C31	Ru2	Ru3	73.00(18)	C28	C27	C26	119.8(8)
C31	Ru2	P2	156.38(19)	C27	C28	C29	121.6(8)
C31	Ru2	C30	35.9(2)	C28	C29	C24	119.1(7)
C31	Ru2	C32	37.1(2)	Ru3	C30	Ru2	77.5(2)
C31	Ru2	C33	63.4(2)	C31	C30	Ru2	71.3(4)
C32	Ru2	Ru3	71.80(16)	C31	C30	Ru3	116.5(5)
C32	Ru2	P2	120.05(17)	C31	C30	C40	120.9(7)
C32	Ru2	C33	35.4(2)	C40	C30	Ru2	125.3(5)
C33	Ru2	Ru3	47.17(17)	C40	C30	Ru3	122.4(5)
C6	Ru3	Ru2	109.5(2)	C30	C31	Ru2	72.8(4)
C6	Ru3	C7	91.5(3)	C30	C31	C32	114.7(6)
C6	Ru3	C30	93.7(3)	C30	C31	C38	125.4(7)
C6	Ru3	C33	167.3(3)	C32	C31	Ru2	74.5(4)
C7	Ru3	Ru2	112.0(2)	C32	C31	C38	119.7(6)

Table S12 Bond Angles for **3**

Atom	Atom	Atom	Angle/°
C7	Ru3	C30	166.0(3)
C7	Ru3	C33	93.8(3)
C8	Ru3	Ru2	136.8(2)
C8	Ru3	C6	97.8(3)
C8	Ru3	C7	99.7(3)
C8	Ru3	C30	92.5(3)
C8	Ru3	C33	92.6(3)
C30	Ru3	Ru2	54.01(19)
C33	Ru3	Ru2	57.8(2)
C33	Ru3	C30	78.6(3)
C17	P1	Ru1	107.9(2)
C17	P1	C18	106.7(3)
C18	P1	Ru1	111.7(2)
C24	P1	Ru1	122.4(2)
C24	P1	C17	104.0(3)
C24	P1	C18	102.9(3)
Ru2	P2	Ru1	123.08(8)
C11	P2	Ru1	99.9(2)
C11	P2	Ru2	111.5(2)
C11	P2	C16	99.9(3)
C16	P2	Ru1	104.8(2)
C38	C31	Ru2	125.5(5)
C31	C32	Ru2	68.3(4)
C33	C32	Ru2	74.7(4)
C33	C32	C31	114.7(6)
C37	C32	Ru2	128.5(5)
C37	C32	C31	125.2(7)
C37	C32	C33	120.0(7)
Ru3	C33	Ru2	75.1(2)
C32	C33	Ru2	69.8(4)
C32	C33	Ru3	115.2(5)
C34	C33	Ru2	129.5(5)
C34	C33	Ru3	127.9(6)
C34	C33	C32	116.4(7)
C35	C34	C33	121.2(7)
C34	C35	C36	121.3(7)
C37	C36	C35	119.7(8)
C36	C37	C32	121.2(8)
C31	C38	C39	110.6(7)
C30	C40	C41	112.7(6)
C44	C43	C42	122.1(10)
C44[1]	C44	C43	125.1(15)

Table S12 Bond Angles for **3**

Atom	Atom	Atom	Angle/°	Atom	Atom	Atom	Angle/°
C16	P2	Ru2	114.4(2)				

Table S13 Torsion Angles for **3**

A	B	C	D	Angle/°	A	B	C	D	Angle/°
Ru1	P1	C17	C16	32.5(5)	C17	P1	C18	C23	-169.9(5)
Ru1	P1	C18	C19	-102.8(6)	C17	P1	C24	C25	63.9(7)
Ru1	P1	C18	C23	72.4(6)	C17	P1	C24	C29	-117.6(6)
Ru1	P1	C24	C25	-173.9(5)	C18	P1	C17	C16	-87.7(5)
Ru1	P1	C24	C29	4.7(7)	C18	P1	C24	C25	-47.3(7)
Ru1	P2	C11	C10	4.4(6)	C18	P1	C24	C29	131.3(6)
Ru1	P2	C11	C12	-179.1(5)	C18	C19	C20	C21	1.8(12)
Ru1	P2	C16	C17	40.6(5)	C19	C18	C23	C22	0.9(11)
Ru1	C9	C10	C11	-5.6(9)	C19	C20	C21	C22	-1.6(12)
Ru1	C9	C10	C14	175.7(5)	C20	C21	C22	C23	1.1(12)
Ru2	P2	C11	C10	-127.2(5)	C21	C22	C23	C18	-0.7(12)
Ru2	P2	C11	C12	49.3(5)	C23	C18	C19	C20	-1.5(11)
Ru2	P2	C16	C17	178.3(4)	C24	P1	C17	C16	163.9(5)
Ru2	C30	C31	C32	-63.3(5)	C24	P1	C18	C19	124.1(6)
Ru2	C30	C31	C38	121.8(7)	C24	P1	C18	C23	-60.8(6)
Ru2	C30	C40	C41	169.0(6)	C24	C25	C26	C27	-0.4(15)
Ru2	C31	C32	C33	-60.3(5)	C25	C24	C29	C28	-2.0(12)

Table S13 Torsion Angles for **3**

A	B	C	D	Angle/°	A	B	C	D	Angle/°
Ru2	C31	C32	C37	122.6(7)	C25	C26	C27	C28	-1.5(15)
Ru2	C31	C38	C39	-177.0(6)	C26	C27	C28	C29	1.6(15)
Ru2	C32	C33	Ru3	-61.5(4)	C27	C28	C29	C24	0.1(14)
Ru2	C32	C33	C34	125.0(6)	C29	C24	C25	C26	2.1(13)
Ru2	C32	C37	C36	-94.6(8)	C30	C31	C32	Ru2	62.3(6)
Ru2	C33	C34	C35	87.2(9)	C30	C31	C32	C33	2.0(9)
Ru3	C30	C31	Ru2	64.9(4)	C30	C31	C32	C37	-175.1(7)
Ru3	C30	C31	C32	1.6(8)	C30	C31	C38	C39	89.4(9)
Ru3	C30	C31	C38	-173.3(6)	C31	C30	C40	C41	-103.0(8)
Ru3	C30	C40	C41	71.3(8)	C31	C32	C33	Ru2	56.8(5)
Ru3	C33	C34	C35	-170.1(5)	C31	C32	C33	Ru3	-4.7(8)
P1	C18	C19	C20	173.6(6)	C31	C32	C33	C34	-178.2(6)
P1	C18	C23	C22	-174.5(6)	C31	C32	C37	C36	176.3(7)
P1	C24	C25	C26	-179.3(7)	C32	C31	C38	C39	-85.3(9)
P1	C24	C29	C28	179.4(6)	C32	C33	C34	C35	2.4(10)
P2	C11	C12	C13	110.5(6)	C33	C32	C37	C36	-0.6(11)
P2	C16	C17	P1	-48.6(6)	C33	C34	C35	C36	-2.3(11)
O9	C9	C10	C11	168.6(7)	C34	C35	C36	C37	0.7(12)
O9	C9	C10	C14	-10.0(10)	C35	C36	C37	C32	0.8(12)
C9	C10	C11	P2	0.1(9)	C37	C32	C33	Ru2	-125.9(6)
C9	C10	C11	C12	-176.1(6)	C37	C32	C33	Ru3	172.5(5)

Table S13 Torsion Angles for **3**

A	B	C	D	Angle/°	A	B	C	D	Angle/°
C9	C10	C14	C15	-72.3(8)	C37	C32	C33	C34	-0.9(10)
C10	C11	C12	C13	-73.3(9)	C38	C31	C32	Ru2	-122.4(7)
C11	P2	C16	C17	-62.5(5)	C38	C31	C32	C33	177.3(6)
C11	C10	C14	C15	109.2(9)	C38	C31	C32	C37	0.2(11)
C14	C10	C11	P2	178.6(5)	C40	C30	C31	Ru2	-120.5(6)
C14	C10	C11	C12	2.4(12)	C40	C30	C31	C32	176.2(6)
C16	P2	C11	C10	111.5(6)	C40	C30	C31	C38	1.2(11)
C16	P2	C11	C12	-72.0(5)	C42	C43	C44	$C44^{1}$	-172.8(17)
C17	P1	C18	C19	14.9(7)					

Table S14 Hydrogen Atom Coordinates (Å×10^4) and Isotropic Displacement Parameters (Å^2×10^3) for **3**

Atom	*x*	*y*	*z*	U(eq)
H12A	4068.71	4004.91	4803.84	38
H12B	3125.37	4935.6	4305.42	38
H13A	1142.18	4263.36	4935.63	69
H13B	1763.34	4681.84	5533.14	69
H13C	2120.91	3462.75	5495.85	69
H14A	4308.17	2274.35	5139.92	45
H14B	5296.09	1355.66	4646.04	45
H15A	3289.06	724.29	4726.23	70

Table S14 Hydrogen Atom Coordinates (Å×10^4) and Isotropic Displacement Parameters (Å^2×10^3) for **3**

**Atom**	***x***	***y***	***z***	**U(eq)**
H15B	2341.2	1619.62	5254.34	70
H15C	3566.36	722.9	5589.47	70
H16A	979.46	5109.52	3529.89	32
H16B	1001.94	5096.25	2614.68	32
H17A	-412.11	4055.04	3314.27	30
H17B	709.23	3449	3863.02	30
H19	-624.37	4673.06	2033.28	44
H20	-1592.73	5194.09	898.26	54
H21	-1205.35	4011.48	-11.42	53
H22	268.56	2340.22	210.61	58
H23	1275.36	1828.82	1352.78	43
H25	-1266.82	2341.48	3151.38	55
H26	-2078.74	916.08	3829.37	77
H27	-468.92	-622.47	4226.2	68
H28	1908.8	-750.93	3901.79	65
H29	2751.95	655.9	3230.58	46
H34	404.66	7062.48	3368.94	41
H35	323.44	7079.38	4702.74	44
H36	2348.18	6985.03	5169.34	51
H37	4465.83	6914.85	4288.45	45

Table S14 Hydrogen Atom Coordinates (Å×10^4) and Isotropic Displacement Parameters (Å^2×10^3) for **3**

**Atom**	***x***	***y***	***z***	**U(eq)**
H38A	7180.6	6702.93	2495.37	48
H38B	6438.49	6567.53	3410.11	48
H39A	6290.41	8536.71	2458.09	92
H39B	7243.52	8087.44	3117.81	92
H39C	5567.1	8396.43	3374.12	92
H40A	6057.81	6778.09	773.43	40
H40B	6925.7	6977.86	1356.01	40
H41A	6625.98	8373.79	309.3	80
H41B	5876.49	8791.9	1144.46	80
H41C	4956.23	8605.85	585.15	80
H42A	8740.31	-688.8	1966.59	148
H42B	7367.96	270.84	2048.83	148
H42C	8882.42	447.33	1978.47	148
H43A	7843.17	-50.26	798.38	120
H43B	8212.83	1003.87	797.89	120
H44A	10568.63	91.45	604.62	118
H44B	10112.64	-889.58	511.15	118
